# Cyclin D1 overexpression induces replication stress and microhomology-mediated end-joining dependence in mantle cell lymphoma

**DOI:** 10.1172/JCI193006

**Published:** 2025-07-03

**Authors:** Jithma P. Abeykoon, Shuhei Asada, Guangli Zhu, Yuna Hirohashi, Lisa Moreau, Divya Iyer, Sirisha Mukkavalli, Kalindi Parmar, Gabriella Zambrano, Lige Jiang, Dongni Yi, Michelle Manske, Kimberly Gwin, Rebecca L. King, James R. Cerhan, Xiaosheng Wu, Zhenkun Lou, Geoffrey I. Shapiro, Thomas Witzig, Alan D’Andrea

**Affiliations:** 1 Division of Hematology, Department of Internal Medicine, Mayo Clinic, Rochester, Minnesota, USA.; 2 Department of Radiation Oncology, Dana-Farber Cancer Institute, Boston, Massachusetts, USA.; 3 Department of Radiation Oncology, The First Affiliated Hospital, Sun Yat-sen University, Guangzhou, China.; 4 Bayer Pharmaceuticals, Cambridge, Massachusetts, USA.; 5 Dr. Kiran C. Patel College of Osteopathic Medicine, Nova Southeastern University, Fort Lauderdale, Florida, USA.; 6 Department of Pathology,; 7 Department of Quantitative Health Sciences, and; 8 Department of Oncology, Mayo Clinic, Rochester, Minnesota, USA.; 9 Department of Medical Oncology, Dana-Farber Cancer Institute, Boston, Massachusetts, USA.

**Keywords:** Cell biology, Hematology, DNA repair, Lymphomas

## Abstract

Oncogene expression can cause replication stress (RS), leading to DNA double-strand breaks (DSBs) that require repair through pathways such as homologous recombination, nonhomologous end-joining, and microhomology-mediated end-joining (MMEJ). Cyclin D1 (encoded by *CCND1*) is a well-known oncoprotein overexpressed in cancer; however, its role in RS is unknown. Using mantle cell lymphoma (MCL) as a naturally occurring model of cyclin D1 overexpression, we examined the impact of cyclin D1 on RS and DSB repair mechanisms. Cyclin D1 overexpression elevated RS, increased DNA damage, especially during mitosis, and caused specific upregulation of MMEJ. Furthermore, cyclin D1 activated polymerase theta (*POLQ*) transcription by binding its promoter loci, driving POLΘ-mediated MMEJ that is essential to withstand cyclin D1–induced RS. Moreover, concurrent ATM deficiency further intensified RS, enhanced *POLQ* expression, and heightened reliance on MMEJ-mediated DNA damage repair. Consequently, inhibition of POLΘ in cyclin D1–overexpressed settings further exacerbated RS, causing single-strand DNA gap accumulations and chromosomal instability, ultimately leading to apoptosis, an effect amplified in ATM-deficient cells. Targeting MMEJ via POLΘ inhibition is therefore an effective strategy in the context of cyclin D1 overexpression and ATM deficiency and may provide a unique therapeutic approach for treating MCL and other malignancies characterized by similar alterations.

## Abstract 2

Cyclin D1 overexpression in cancer induces replication stress and DNA damage, forcing cells to rely on specific repair pathways that can be targeted for therapy.

## Graphical abstract

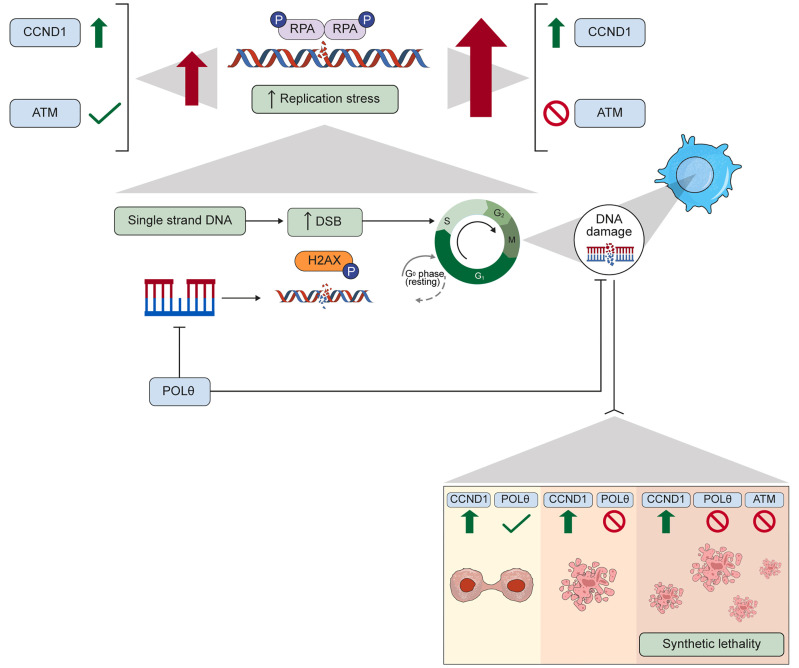

## Introduction

Despite the inherent accuracy of DNA replication, various exogenous and endogenous stresses experienced by cells, collectively termed “replication stress” (RS), challenge the fidelity of this highly sophisticated machinery. RS results in replication fork slowing, increased single-stranded DNA gaps, reduced replication fidelity, and the generation of DNA double-strand breaks (DSBs) (1–3). DSBs can be lethal if not repaired by pathways such as homologous recombination (HR), nonhomologous end-joining (NHEJ), or microhomology-mediated end-joining (MMEJ) (4). These DSB repair pathways are highly cell cycle regulated and occur during the S/G_2_ phases, G_1_ phase, or mitosis, respectively (5, 6).

Oncogene overexpression, such as *c-Myc* (7), *K-RAS* (8), and *BCL-2* (9), among many others, with its resultant high proliferation rate, is a major source of RS. Heightened RS is proportional to the generation of DSBs, which in turn can overwhelm the DSB repair pathways and produce a high DSB burden (3). Furthermore, oncogene expression can also perturb cell cycle checkpoints, causing unrepaired DSB to traverse the cell cycle without activation of cell cycle arrest (10–15). Therefore, unrepaired DSBs can accumulate in mitosis, a time when the HR and NHEJ pathways are inactive (16, 17). Consequently, cancer cells driven by oncogene-induced proliferation rely heavily on the MMEJ pathway for DSB repair.

Cyclin D1 (encoded by *CCND1*) is frequently overexpressed in cancers by a variety of mechanisms (18). In addition to gene amplification, chromosomal rearrangements may occur, such as the t(11;14) translocation, occurring nearly universally in mantle cell lymphoma (MCL) (19, 20). Overexpression of cyclin D1 also results from the activation of upstream signaling driven by the MAPK and NF-κB pathways (21–26). Cyclin D1 regulates cell cycle progression; its overexpression allows transcription of genes involved in G_1_ and S phase progression and permits DNA replication (27–31). In cells with cyclin D1 overexpression, the transition from the G_1_ to S phase is accelerated, leading to increased DNA replication and cell proliferation.

Although cyclin D1 is known to drive cells to enter the S phase and initiate DNA replication, little is known about how cancer cells adapt to the resultant RS. Cyclin D1 overexpression is nearly ubiquitous in MCL, and approximately 10%–20% of other non-Hodgkin lymphomas (NHLs) also exhibit this characteristic phenotype (32, 33). Moreover, MCL is known to harbor high chromosomal and genomic instability compared with other NHLs (34, 35). While progress has been made in the treatment of MCL, the development of relapsed, refractory disease is common and remains incurable, representing an area of high unmet medical need. We therefore utilized MCL as a model system to explore the effects of cyclin D1 overexpression on the induction of RS and DSB repair pathways. We found that ataxia-telangiectasia mutated (ATM) deficiency, the second most common genomic alteration in MCL observed in 40%–50% of cases (36–38), exacerbated CCND1-induced RS, with accumulation of DNA damage in the mitotic phase of the cell cycle, increasing reliance of MCL cells on MMEJ. Cyclin D1 binds to the *POLQ* gene promoter loci, resulting in increased protein expression of polymerase theta (POLΘ), the primary DNA polymerase of MMEJ (6), to promote the maintenance of genomic integrity in MCL. This increased *POLQ* expression is further increased with concurrent ATM deficiency with cyclin D1 overexpression. Consequently, cyclin D1–expressing MCL cells, particularly those with concomitant ATM deficiency, are vulnerable to POLΘ inhibition, unveiling a potential biomarker-driven treatment option for this patient population.

## Results

### Cyclin D1 increases DNA RS and associated DSBs.

**Figure 1:**
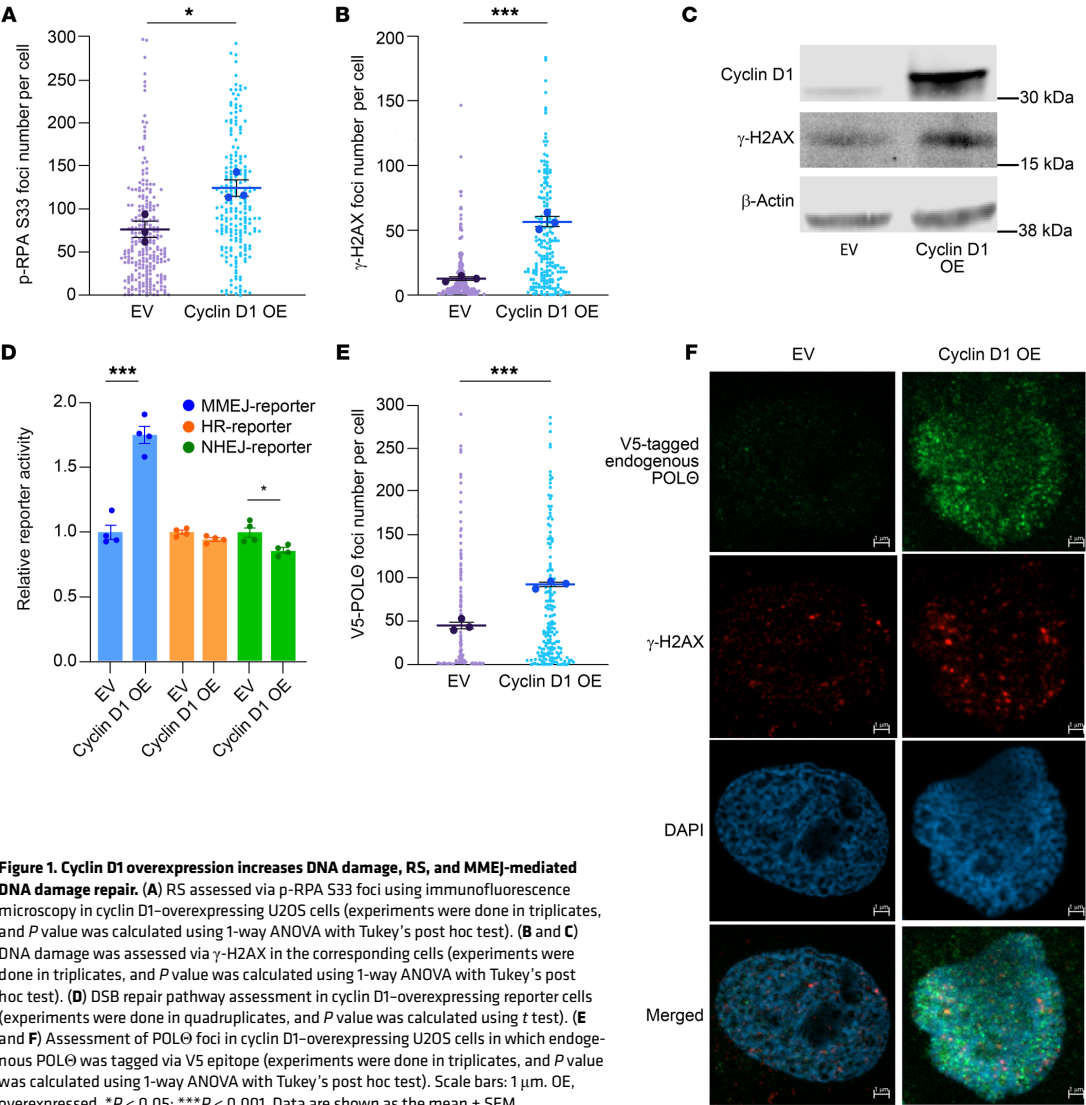
Cyclin D1 overexpression increases DNA damage, RS, and MMEJ-mediated DNA damage repair. (**A**) RS assessed via p-RPA S33 foci using immunofluorescence microscopy in cyclin D1–overexpressing U2OS cells (experiments were done in triplicates, and *P* value was calculated using 1-way ANOVA with Tukey’s post hoc test). (**B** and **C**) DNA damage was assessed via γ-H2AX in the corresponding cells (experiments were done in triplicates, and *P* value was calculated using 1-way ANOVA with Tukey’s post hoc test). (**D**) DSB repair pathway assessment in cyclin D1–overexpressing reporter cells (experiments were done in quadruplicates, and *P* value was calculated using *t* test). (**E** and **F**) Assessment of POLΘ foci in cyclin D1–overexpressing U2OS cells in which endogenous POLΘ was tagged via V5 epitope (experiments were done in triplicates, and *P* value was calculated using 1-way ANOVA with Tukey’s post hoc test). Scale bars: 1 μm. OE, overexpressed. *P < 0.05; ***P < 0.001. Data are shown as the mean ± SEM.

Before characterizing RS and critical DNA repair pathways in cyclin D1 overexpressed MCL cells, we sought to understand the effects of cyclin D1 overexpression in an isogeneic background. *CCND1* cDNA was lentivirally transduced into U2OS cells, and the cells were assessed for RS markers and DNA damage (Figure 1, A–C). Indeed, cyclin D1–overexpressing U2OS cells exhibited elevated levels of phospho-RPA S33 (p-RPA), a well-established marker for RS (39), compared with empty vector–transduced (EV-transduced) control cells (Figure 1A). This increase in p-RPA correlated with increased DNA DSBs, as evaluated by γ-H2AX (Figure 1, B and C). Higher RS and unrepaired DNA damage were evident in cyclin D1–overexpressing cells, yet they proliferated more rapidly compared with EV-transduced cells (Supplemental Figure 1A; supplemental material available online with this article; https://doi.org/10.1172/JCI193006DS1). This suggests that cyclin D1–overexpressing cells may depend more heavily on DSB repair pathways to cope with RS, ultimately preventing cell death and facilitating rapid cell growth.

### Cyclin D1 overexpression specifically increases MMEJ pathway activity.

To study the activation of DNA DSB repair pathways in response to cyclin D1 overexpression, we used U2OS cell–based standardized reporter assays to assess the HR, NHEJ, and MMEJ pathways (40–42). Cyclin D1 was overexpressed in the reporter cells using a lentiviral construct and validated by immunoblotting (Supplemental Figure 1B). These cells exhibited significantly increased MMEJ activity without enhancement of NHEJ or HR, suggesting that cyclin D1–overexpressing cells have specific dependency on MMEJ (Figure 1D).

Given that MMEJ is the major DSB repair pathway activated by cyclin D1 overexpression, we next assessed the localization and accumulation of POLΘ in cyclin D1–overexpressing U2OS cells. We generated a U2OS clone in which the biallelic N-terminus of the endogenous *POLQ* locus was tagged by a V5 epitope and validated the V5-tagged POLΘ cells through immunoblotting and assessing foci with CRISPR-induced depletion of *POLQ* (Supplemental Figure 1, C–E). Indeed, when cyclin D1 was overexpressed in these cells, there was an accumulation of POLΘ foci (Figure 1E), and some colocalized with γ-H2AX foci (Figure 1F). Therefore, the increased DNA damage resulting from cyclin D1 overexpression activated POLΘ recruitment and MMEJ to promote physiologic DSB repair.

### MCL cells accumulate unrepaired DNA damage in mitosis, leading to cellular dependence on MMEJ.

**Figure 2:**
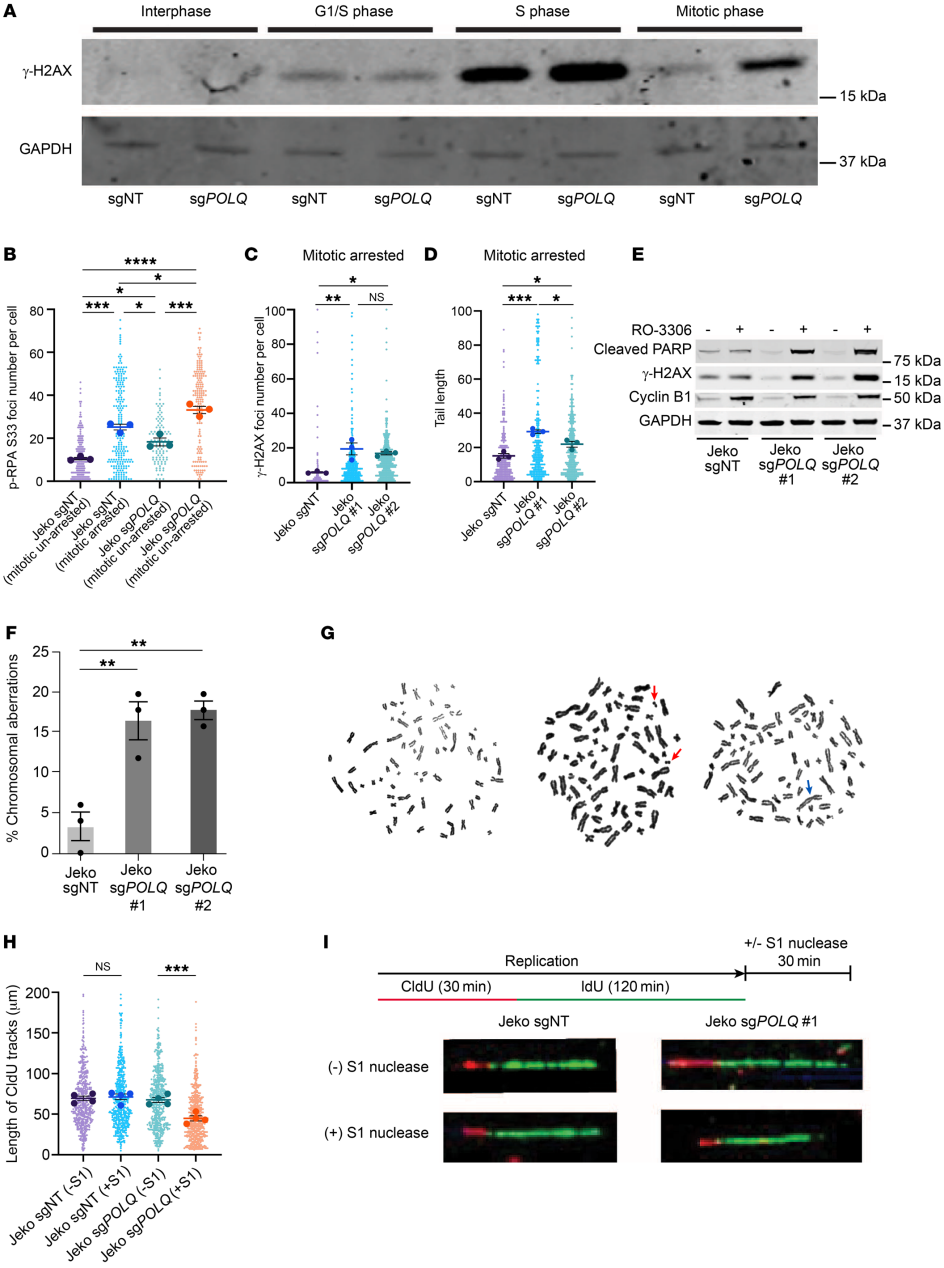
MCL cells rely on POLΘ and MMEJ-mediated DSB repair to repair mitotic DNA DSBs and to mitigate detrimental RS. (**A**) Assessment of DNA damage as the MCL cell traverses through the different phases of the cell cycle in POLΘ-deficient and -proficient conditions. (**B**) Assessment of RS through p-RPA S33 foci in unsynchronized and mitotic synchronized cells based on POLΘ proficiency (experiments were done in triplicates, and *P* value was calculated using 1-way ANOVA with Tukey’s post hoc test). (**C** and **D**) Assessment of mitotic DNA damage in POLΘ-proficient and -deficient MCL cells via γ-H2AX foci and comet assay, respectively (experiments were done in triplicates, and *P* value was calculated using 1-way ANOVA with Tukey’s post hoc test). (**E**) Assessment of apoptotic marker (cleaved PARP) and DNA damage (γ-H2AX) in POLΘ-proficient and -deficient mitotically synchronized (validated by cyclin B1 expression) and unsynchronized MCL cells. (**F** and **G**) Assessment of chromosomal stability (red arrows show double minute chromosomes, and blue arrow shows a dicentric chromosome) in POLΘ-deficient and -proficient MCL cells (experiments were done in triplicates, and *P* value was calculated using 1-way ANOVA with Tukey’s post hoc test). (**H** and **I**) Prevalence of single-stranded DNA in MCL cells with and without POLΘ assessed through DNA fiber S1 nuclease assay (experiments were done in quadruplicates, and *P* value was calculated using 1-way ANOVA with Tukey’s post hoc test). *P < 0.05; **P < 0.01; ***P < 0.001; ****P < 0.0001. Data are shown as the mean ± SEM.

We next studied RS, DNA damage, and DNA repair in MCL cells carrying the t(11;14) translocation leading to high cyclin D1 expression (43). To delineate the importance of the MMEJ pathway in MCL, we used Jeko cells that carry a 3xFLAG tag at the N-terminus of the endogenous *POLQ* gene and generated isogeneic *POLQ*-deficient cells using CRISPR-Cas9 technology (Supplemental Figure 2A). This allowed us to assess the role of MMEJ in MCL by profiling replication-associated physiologic DNA damage in the presence or absence of POLΘ following the synchronization of cells at the G_1_/S boundary (Supplemental Figure 2B). Physiologic DNA damage associated with DNA replication begins as the cells enter S phase (Figure 2A). Importantly, this DNA damage was repaired in POLΘ*-*proficient Jeko cells after they traversed S phase and entered the mitotic phase of the cell cycle. However, unrepaired DNA damage persisted in mitosis in the absence of POLΘ. This suggests that in MCL cells, MMEJ is critical for resolving replication-associated mitotic DNA damage. In the absence of POLΘ, unrepaired DNA damage accumulates in mitosis, leading to genomic aberrations and cell death (6, 44).

In addition, we assessed RS by probing for p-RPA and DNA damage through γ-H2AX expression and by using a comet assay in *POLQ* control and *POLQ-*deficient MCL cells. In the absence of POLΘ, the expression of p-RPA and γ-H2AX significantly increased, with the induction of comet tails. These effects were further enhanced when cells were synchronized in mitosis (Figure 2, B–D). MMEJ therefore plays a critical role in MCL in mitigating replication-associated DNA damage, a process that becomes critical in mitosis. Moreover, POLΘ-deficient MCL cells exhibited increased expression of apoptotic markers (e.g., cleaved PARP) (45) along with increased γ-H2AX when synchronized in mitosis (Figure 2E and Supplemental Figure 2C). These data indicate that the inhibition of MMEJ in cyclin D1–overexpressing cells compromises survival, with death likely occurring predominantly in mitosis.

### The absence of POLΘ causes chromosomal instability in MCL.

Since the accumulation of unrepaired DNA damage in mitosis is more evident with POLΘ deficiency in high cyclin D1–expressing MCL cells, we asked whether reduced POLΘ expression is also associated with chromosomal instability. To address this question, we assessed the number of chromosomal aberrations at a given time in isogeneic WT and POLΘ-deficient MCL cells. Indeed, in Jeko cells, the absence of POLΘ caused a significant increase in chromosomal aberrations (double minute chromosomes and dicentric chromosomes) correlating with the increased levels of RS, unrepaired DNA damage during mitosis, and increased expression of apoptotic markers (Figure 2, E–G).

### The absence of POLΘ in MCL causes RS by increasing single-strand DNA gaps and confers sensitivity to ATR or PARP inhibition.

Given the increased RS in MCL cells lacking POLΘ, we assessed the effects of POLΘ deficiency on DNA replication using DNA fiber assays. POLΘ-deficient MCL cells demonstrated a significant increase in single-strand DNA gaps compared with POLΘ-proficient isogeneic cells (Figure 2, H and I). Single-strand DNA gaps exacerbate RS and are detrimental to cell proliferation and survival, suggesting a mechanism for RS-induced apoptosis in POLΘ-deficient MCL cells (2, 46).

We also looked at the susceptibility of POLΘ-deficient cells to ataxia-telangiectasia and Rad3-related (ATR) inhibition, a key kinase known to promote the resolution of RS (2). Confirming the heightened RS in POLΘ-deficient MCL cells, these cells showed increased sensitivity to ATR inhibition compared with POLΘ-proficient MCL cells (Supplemental Figure 2D). We also asked whether POLΘ deficiency conferred PARP inhibitor sensitivity. Parental Jeko cells are HR repair proficient and PARP inhibitor resistant (47). However, PARP inhibitors can increase RS by trapping PARP on DNA (48), accelerating replication fork speed (49) and impeding the maturation of nascent DNA strands (50). Indeed, the PARP inhibitor olaparib reduced the viability of POLΘ-deficient cells, whereas control cells were unaffected (Supplemental Figure 2E). These results further confirm the increased RS experienced by HR-proficient MCL cells in the absence of POLΘ that exceeds a lethal threshold upon challenge with ATR or PARP inhibition.

### Cyclin D1 promotes MMEJ by directly upregulating POLQ promoter activity.

**Figure 3:**
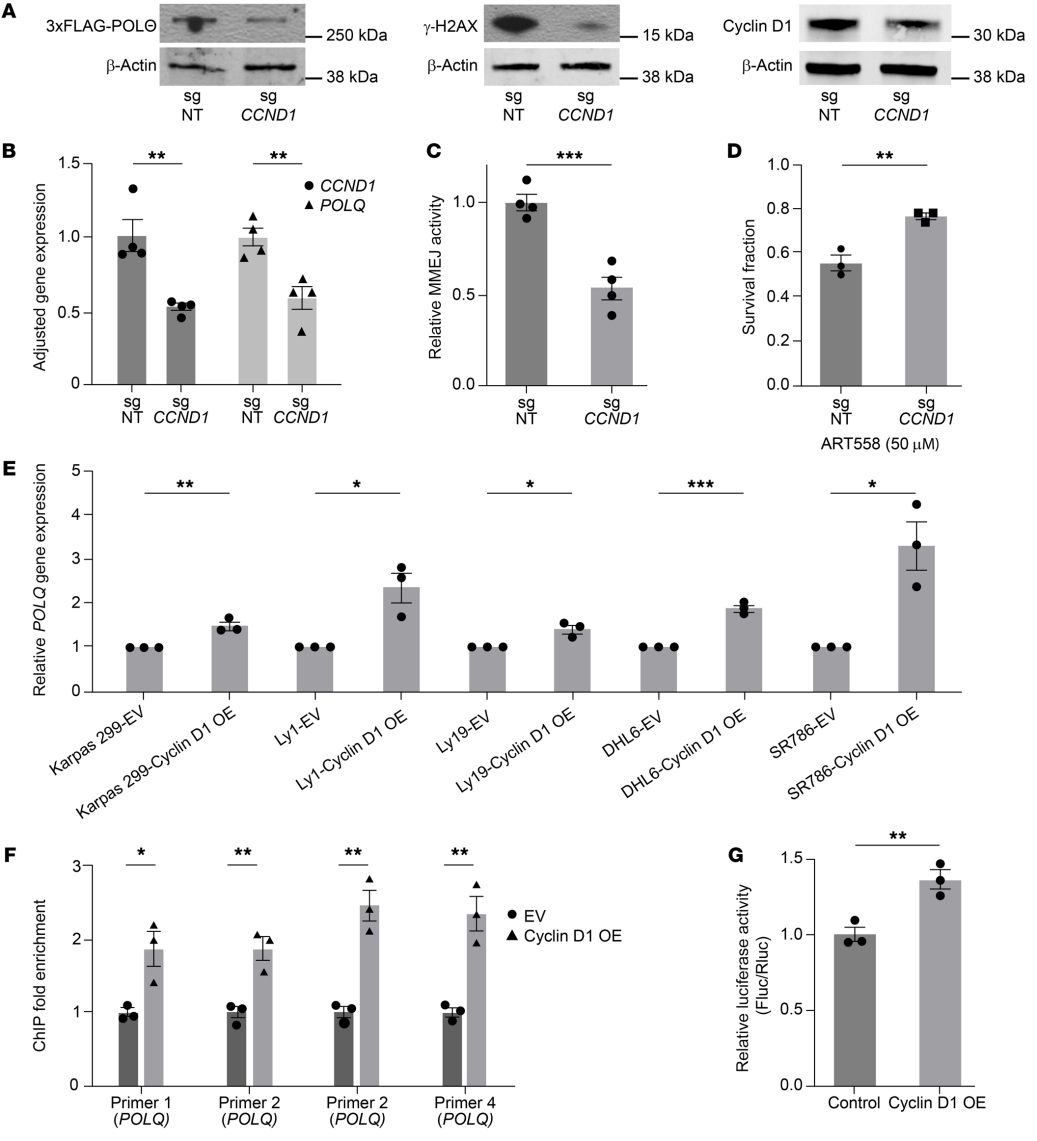
Cyclin D1 overexpression specifically increases the expression of *POLQ* through binding to the *POLQ* promoter, which leads to increased MMEJ-mediated DNA damage repair. (**A**) Assessment of *POLQ* expression and DNA damage (γ-H2AX) in MCL cells (Jeko) when cyclin D1 expression is decreased through CRISPRi technology. (**B**) Quantification of *POLQ* and *CCND1* transcription in Jeko cells with decreased cyclin D1 expression (experiments were done in quadruplicates, and *P* value was calculated using *t* test). (**C**) Assessment of MMEJ-mediated DNA damage repair in Jeko cells with decreased cyclin D1 expression (experiments were done in quadruplicates, and *P* value was calculated using *t* test). (**D**) Assessment of sensitivity to POLΘ inhibition (ART558) in Jeko cells with decreased cyclin D1 expression (experiments were done in triplicates, and *P* value was calculated using *t* test). (**E**) Assessment of *POLQ* expression with lentiviral-mediated cyclin D1 overexpression in multiple NHL cell lines belonging to T cell lymphoma (SR786 and Karpas 299) and diffuse large B cell lymphoma (Ly1, Ly19, and DHL6) (experiments were done in triplicates, and *P* value was calculated using *t* test). (**F**) Evaluation of cyclin D1 binding to the *POLQ* promoter region through ChIP in HA-tagged cyclin D1–overexpressing MCL cells (Jeko cells with endogenous cyclin D1 expression stably decreased) (experiments were done in triplicates, and *P* value was calculated using 1-way ANOVA with Tukey’s post hoc test). (**G**) Assessment of the transcriptional activity of the *POLQ* promoter using a luciferase assay in cells with cyclin D1 overexpression (experiments were done in triplicates, and *P* value was calculated using 1-way ANOVA with Tukey’s post hoc test). Fluc, Firefly luciferase; Rluc, Renilla luciferase; OE, overexpressed. *P < 0.05; **P < 0.01; ***P < 0.001. Data are shown as the mean ± SEM.

Since *CCND1* is an essential gene for MCL, its CRISPR knockout is lethal at the cellular level. Therefore, we used CRISPR interference (CRISPRi) technology to stably decrease the expression of cyclin D1 (51). As depicted in Figure 3A, we generated a stable knockdown of cyclin D1 in Jeko cells, in which the endogenous *POLQ* gene is tagged with 3xFLAG. The decreased expression of cyclin D1 had a minimal effect on cell cycle progression (Supplemental Figure 3A). Interestingly, we observed that decreased cyclin D1 expression also resulted in decreased POLΘ protein and *POLQ* mRNA expression and decreased physiologic DNA damage (Figure 3, A and B). Similarly, engineered MCL cells carrying the MMEJ reporter confirmed that decreased expression of cyclin D1 reduced MMEJ-mediated DSB repair (Figure 3C). Cells with reduced cyclin D1 expression were also less sensitive to pharmacologic POLΘ inhibition (Figure 3D).

To confirm the results obtained in the MCL cells, cyclin D1 was ectopically overexpressed in other NHL cell lines, followed by measurement of *POLQ* expression via quantitative PCR (qPCR). As predicted, a significant increase in *POLQ* transcripts was observed in other NHL cell lines overexpressing cyclin D1 (Figure 3E), suggesting that our results may apply to other malignancies harboring cyclin D1 overexpression.

Following observing increased *POLQ* gene expression when cyclin D1 is overexpressed, we investigated whether cyclin D1 directly binds to the *POLQ* promoter (52). To explore this possibility, we overexpressed HA-tagged cyclin D1 in Jeko cells with knocked-down endogenous cyclin D1 (Supplemental Figure 3B) and in U2OS cells (Supplemental Figure 3C) and performed ChIP with an anti-HA antibody, followed by qPCR. The ATM gene was utilized as a negative control because alterations in cyclin D1 expression did not affect ATM gene expression in either U2OS or Jeko cells (Supplemental Figure 3, D and E). We found that cyclin D1 directly binds to the *POLQ* promoter (Figure 3F and Supplemental Figure 3, F and G). Subsequently, we determined the transcriptional activity of the *POLQ* promoter using a luciferase assay. After normalizing the transfection efficiency with Renilla activity, *POLQ* promoter–driven Firefly luciferase activity was significantly increased in HEK cells when cyclin D1 was overexpressed (Figure 3G). These data indicate that cyclin D1 binds to the *POLQ* promoter and enhances its transcriptional activity, leading to upregulation of *POLQ* gene expression.

### Enhancement of RS by concomitant depletion of ATM.

**Figure 4:**
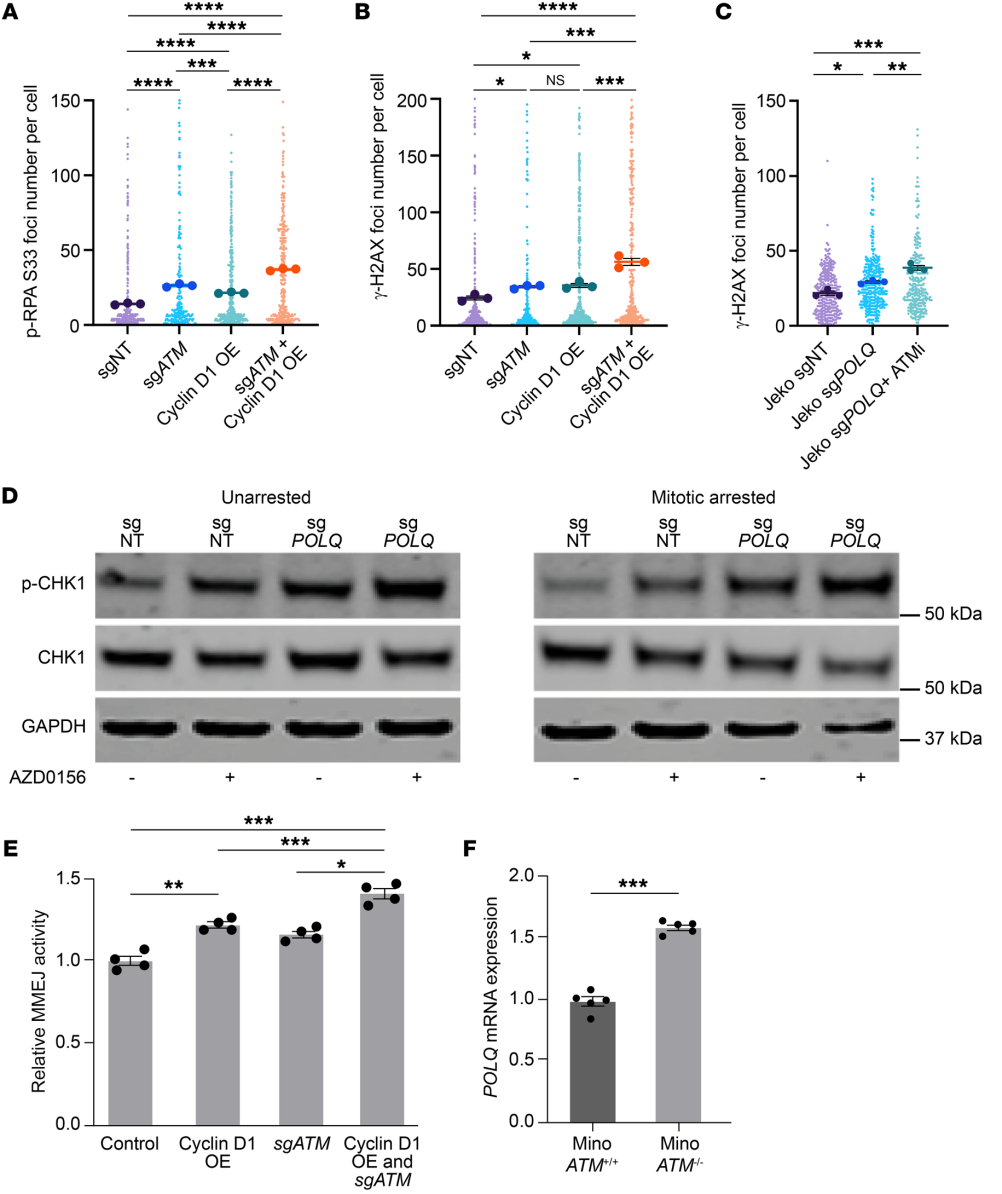
Concurrent deficiency in ATM with cyclin D1 overexpression augments RS, mitotic DNA damage, and MMEJ-mediated DNA damage repair. (**A**) RS assessment via p-RPA S33 foci and (**B**) DNA damage assessment via γ-H2AX in cyclin D1–overexpressing and ATM-deficient U2OS cells (experiments were done in triplicates, and *P* value was calculated using 1-way ANOVA with Tukey’s post hoc test). (**C**) Assessment of DNA damage via γ-H2AX in mitotically synchronized POLΘ-deficient Jeko cells with ATM inhibition (1 μM AZD0156) after mitotic synchronization using RO-3306 (experiments were done in triplicates, and *P* value was calculated using 1-way ANOVA with Tukey’s post hoc test). (**D**) Assessment of RS through a validated marker (phosphorylated CHK1) in POLΘ-proficient and -deficient Jeko cells with and without ATM inhibition in unsynchronized and mitotically synchronized cells using RO-3306 (lanes were run in the same gel but displayed separately to facilitate comparison within the unsynchronized and mitotic arrested groups). (**E**) MMEJ-mediated DNA DSB repair assessment through a validated reporter in cyclin D1–overexpressed and ATM-deficient backgrounds in U2OS reporter cells (experiments were done in quadruplicates, and *P* value was calculated using 1-way ANOVA with Tukey’s post hoc test). (**F**) Assessment of *POLQ* mRNA expression in isogeneic Mino cell line with and without ATM deficiency (experiments were done in quintuplets, and *P* value was calculated using *t* test). OE, overexpressed; ATMi, ATM inhibitor. *P < 0.05; **P < 0.01; ***P < 0.001; ****P < 0.0001. Data are shown as the mean ± SEM.

Approximately 45% of MCLs harbor ATM deficiency (36–38, 53). ATM regulates the DNA damage response, and its absence may reroute more DSB repair to the MMEJ pathway (54). Given this clinical and therapeutic relevance, we determined whether ATM deficiency could further increase RS and DSB repair pathway dependency, specifically in cyclin D1–overexpressing cells. Indeed, ATM deficiency in cyclin D1–overexpressing U2OS cells exacerbated RS, as assessed by increased p-RPA (Figure 4A). As expected, the increased RS was associated with increased γ-H2AX expression (Figure 4B).

We next validated this finding in MCL using our isogeneic Jeko cells treated with an ATM inhibitor. When POLΘ-deficient Jeko cells were treated with the ATM inhibitor AZD0156 (55), a significant increase in unrepaired mitotic DNA damage was observed compared with vehicle-treated WT and POLΘ-deficient cells (Figure 4C). Furthermore, a significant increase in p-CHK1, a validated biomarker for RS (2), was detected when POLΘ-deficient Jeko cells were treated with AZD0156; this increased p-CHK1 was more evident when cells were synchronized in mitosis (Figure 4D).

Using the U2OS cells engineered with DNA repair pathway reporters, we assessed HR, NHEJ, and MMEJ activity in cyclin D1–overexpressing cells in both ATM-proficient and -deficient backgrounds (Supplemental Figure 4, A–C). The upregulation of the MMEJ pathway that occurred with cyclin D1 overexpression was further enhanced by concomitant ATM deficiency, suggesting an increased dependency of these cells on MMEJ when ATM is absent (Figure 4E). The activity of the HR and NHEJ repair pathways was not increased with cyclin D1 overexpression and ATM deficiency, suggesting that these co-occurring alterations uniquely upregulate MMEJ, dictating MMEJ dependence for DSB repair (Supplemental Figure 4, I and J).

We then assessed the *POLQ* expression in MCL in relation to ATM alteration using isogeneic Mino cells. In ATM-deficient Mino cells, *POLQ* expression significantly increased when compared with ATM-proficient cells (Figure 4F and Supplemental Figure 4D). This observation was further confirmed in cyclin D1–overexpressing U2OS cells with and without ATM deficiency (Supplemental Figure 4, A and E). After confirming in isogeneic cell lines, we then studied *POLQ* expression in primary MCL cells. The *POLQ* expression was increased in primary ATM-deficient MCL cells, which was further confirmed by a publicly available MCL gene expression dataset that showed significantly increased *POLQ* expression with no change to *CCND1* expression levels in ATM-mutated MCL compared with ATM-WT MCL (Supplemental Figure 4, F–H) (56). Taken altogether, these data indicate that in cells with cyclin D1 overexpression, especially in MCL, ATM deficiency further leads to increased expression of *POLQ* compared with ATM-proficient cells.

**Figure 5:**
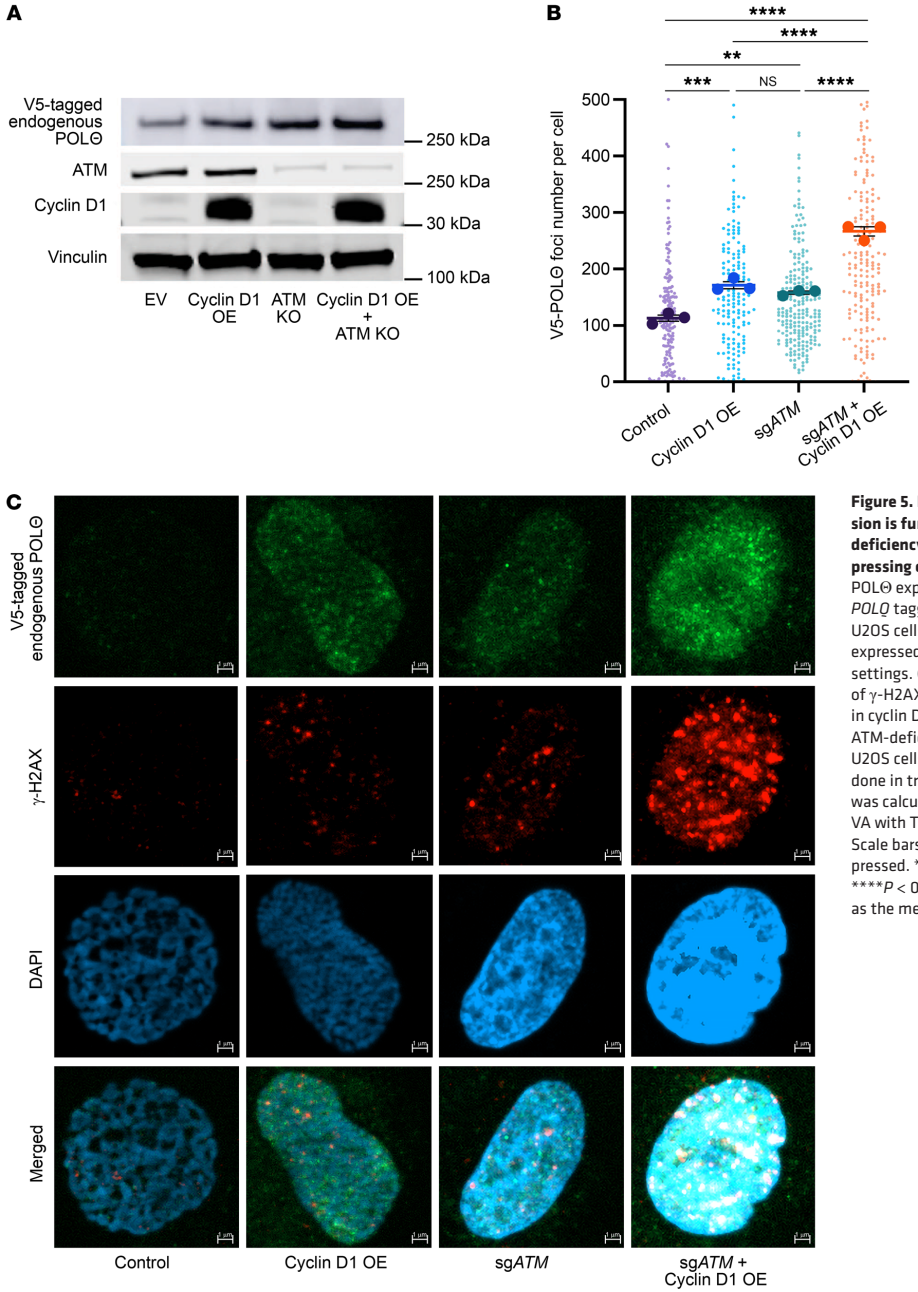
POLΘ protein expression is further increased by ATM deficiency in cyclin D1–overexpressing cells. (**A**) Evaluation of POLΘ expression (endogenous *POLQ* tagged with V5 epitope) in U2OS cells with cyclin D1–overexpressed and ATM-deficient settings. (**B** and **C**) Assessment of γ-H2AX foci and POLΘ foci in cyclin D1–overexpressed and ATM-deficient background in U2OS cells (experiments were done in triplicates, and *P* value was calculated using 1-way ANOVA with Tukey’s post hoc test). Scale bars: 1 μm. OE, overexpressed. **P < 0.01; ***P < 0.001; ****P < 0.0001. Data are shown as the mean ± SEM.

Consistent with increased *POLQ* gene expression when ATM is deficient in the background of cyclin D1 overexpression, POLΘ protein expression in cyclin D1–overexpressing cells was further enhanced by ATM deficiency (Figure 5A). Furthermore, the accumulation of POLΘ as foci into replication-induced DNA damage sites was significantly increased with concomitant cyclin D1 overexpression and ATM deficiency compared with cells with monogenic altered counterparts (Figure 5, B and C). These data suggest that ATM deficiency exacerbates RS in cyclin D1–overexpressing cells, with consequent DNA damage causing increased MMEJ dependence.

### Genetic and pharmacologic MMEJ pathway disruption has antiproliferative effects in MCL cells.

**Figure 6:**
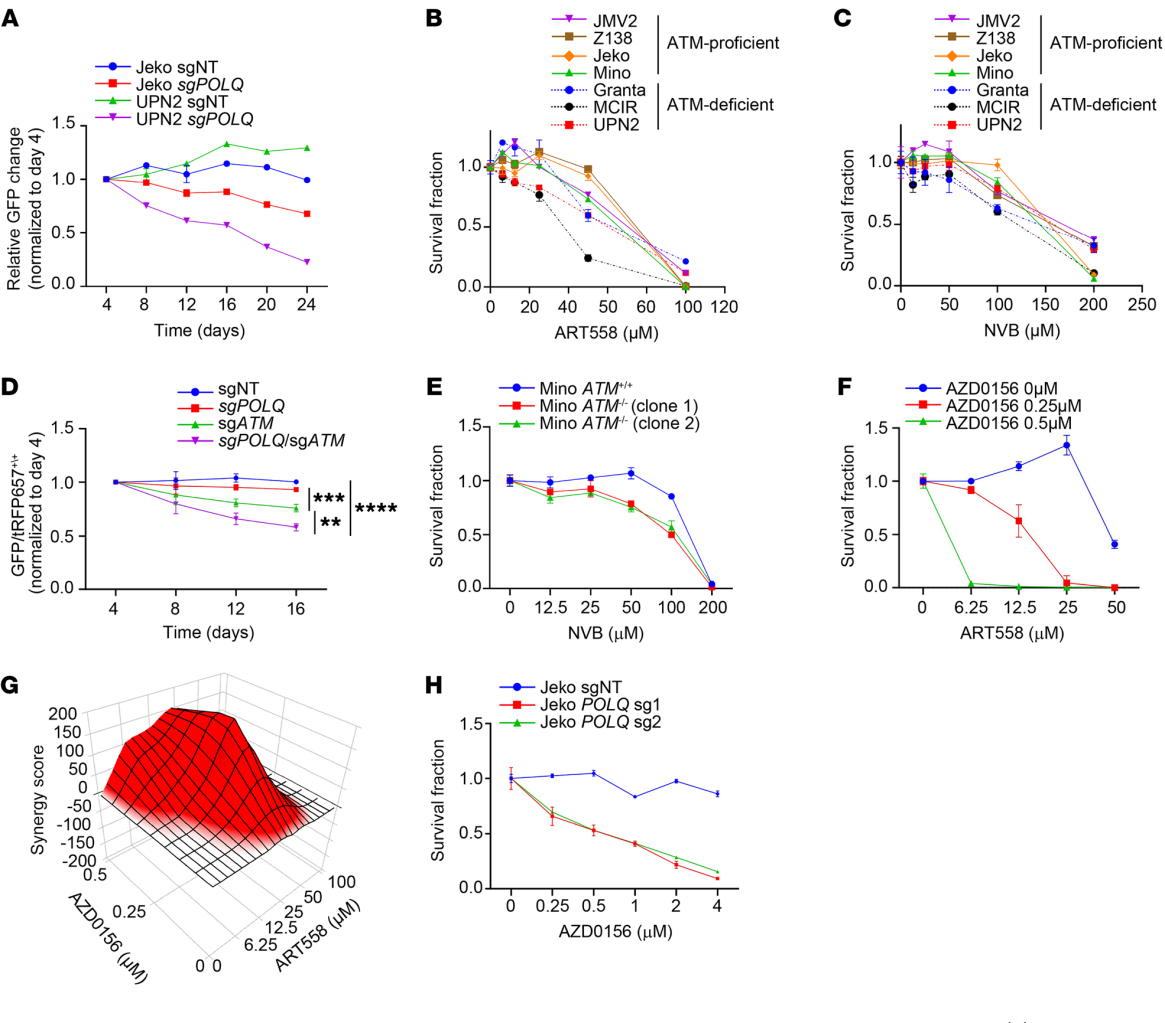
POLΘ inhibition induces a significant antitumor effect in MCL and is augmented by concurrent ATM deficiency. (**A**) Assessment of proliferation rate and viability using a competitive assay in 2 MCL cell lines (Jeko-ATM proficient and UPN2-ATM deficient) with genetic depletion of *POLQ* (experiments were done in triplicates). (**B** and **C**) Cell viability assessment of MCL cell lines using 2 POLΘ inhibitors, ART558 and NVB (experiments were done in triplicates). (**D**) Assessment of antiproliferative and apoptotic effect with *POLQ* and *ATM* genetic depletion using Jeko cell line (experiments were done in quadruplicates, and *P* value was calculated by 2-way mixed-model ANOVA). (**E**) Graph illustrating the sensitivity to POLΘ inhibition by NVB in ATM-deficient and -proficient Mino cells (experiments were done in triplicates). (**F** and **G**) Assessment of cell killing and synergy (Bliss synergy score of 72.91) in Jeko cells with concurrent inhibition of ATM using AZD0156 and POLΘ with ART558 (experiments were done in triplicates). (**H**) Genetically depleted *POLQ* in Jeko cells treated with AZD0156 to assess cell killing in *POLQ*-deficient and -proficient backgrounds (experiments were done in triplicates). **P < 0.01; ***P < 0.001; ****P < 0.0001. Data are shown as the mean ± SD.

Next, we explored targeting POLΘ as a therapeutic strategy in MCL. To assess the impact of POLΘ on cell proliferation and survival, we conducted a competitive proliferation assay in different MCL cell lines after knocking out *POLQ*. *POLQ* genetic depletion caused dampening of cell proliferation in all cell lines tested, with the Granta and UPN2 cell lines being more sensitive compared with the Jeko, JVM-2, and Z138 cell lines (Figure 6A and Supplemental Figure 5A). Subsequently, we used 2 independent POLΘ inhibitors, novobiocin (NVB), an inhibitor of the helicase domain (57), and ART558, an inhibitor of the polymerase domain (58), to validate this antitumor effect in multiple MCL cell lines (Figure 6, B and C). All MCL cell lines were sensitive to both POLΘ inhibitors; again, Granta and UPN2, as well as MCIR cells, which are ATM-deficient MCL cell lines, demonstrated relatively more sensitivity. Consistent with our observations of increased RS and MMEJ pathway dependence when ATM is depleted in cyclin D1–overexpressing cells, ATM-deficient cell lines were hypersensitive to POLΘ inhibition (Supplemental Figure 5B).

To assess the effect of ATM inhibition in a POLΘ-deficient background in MCL, we performed a competitive assay using isogeneic cells with *ATM* and *POLQ* deficiency. Indeed, as shown in Figure 6D, *POLQ* depletion, but not *ATM* depletion, significantly decreased the proliferation of Jeko cells. The simultaneous depletion of *ATM* with *POLQ* significantly increased the antiproliferative and pro-apoptotic effects compared with *POLQ* depletion alone. We also generated isogeneic ATM-depleted MCL cells in the Mino cell line, an *ATM*-WT cell line. ATM-deficient Mino cells were more sensitive to pharmacologic inhibition of POLΘ with NVB than ATM-proficient Mino cells (Figure 6E). Furthermore, cotreatment of ATM-proficient Jeko cells with the ATM inhibitor AZD0156 and the POLΘ inhibitor ART558 caused synergistic antitumor activity (Figure 6, F and G; average synergy Bliss score of 72.91). Conversely, isogeneic *POLQ-*depleted Jeko cells were more sensitive to AZD0156-mediated ATM inhibition than *POLQ*-proficient Jeko cells (Figure 6H).

In addition to validating the enhanced antitumor effect with *POLQ* depletion in cyclin D1–overexpressing and ATM-deficient MCLs, we also recapitulated this effect using U2OS cells. Indeed, cyclin D1–overexpressing cells were more sensitive to POLΘ inhibition by ART558 and NVB. Additionally, pharmacologic POLΘ inhibition had a greater antitumor effect in cyclin D1–overexpressing cells lacking ATM compared with cyclin D1-overexpressing cells that were ATM proficient (Supplemental Figure 5, C and D). Taken together, our data indicate that ATM-deficient MCL cells are especially sensitive to POLΘ inhibition. These results may broadly apply to other cyclin D1–overexpressing tumor cells that are also ATM deficient, as a similar phenotype was also seen in U2OS cells, an osteosarcoma cell line. In contrast, cyclin D1–overexpressing tumor cells that are ATM proficient may be highly vulnerable to combined POLΘ and ATM inhibition.

### POLΘ inhibition produces significant antitumor effects in MCL in vivo.

**Figure 7:**
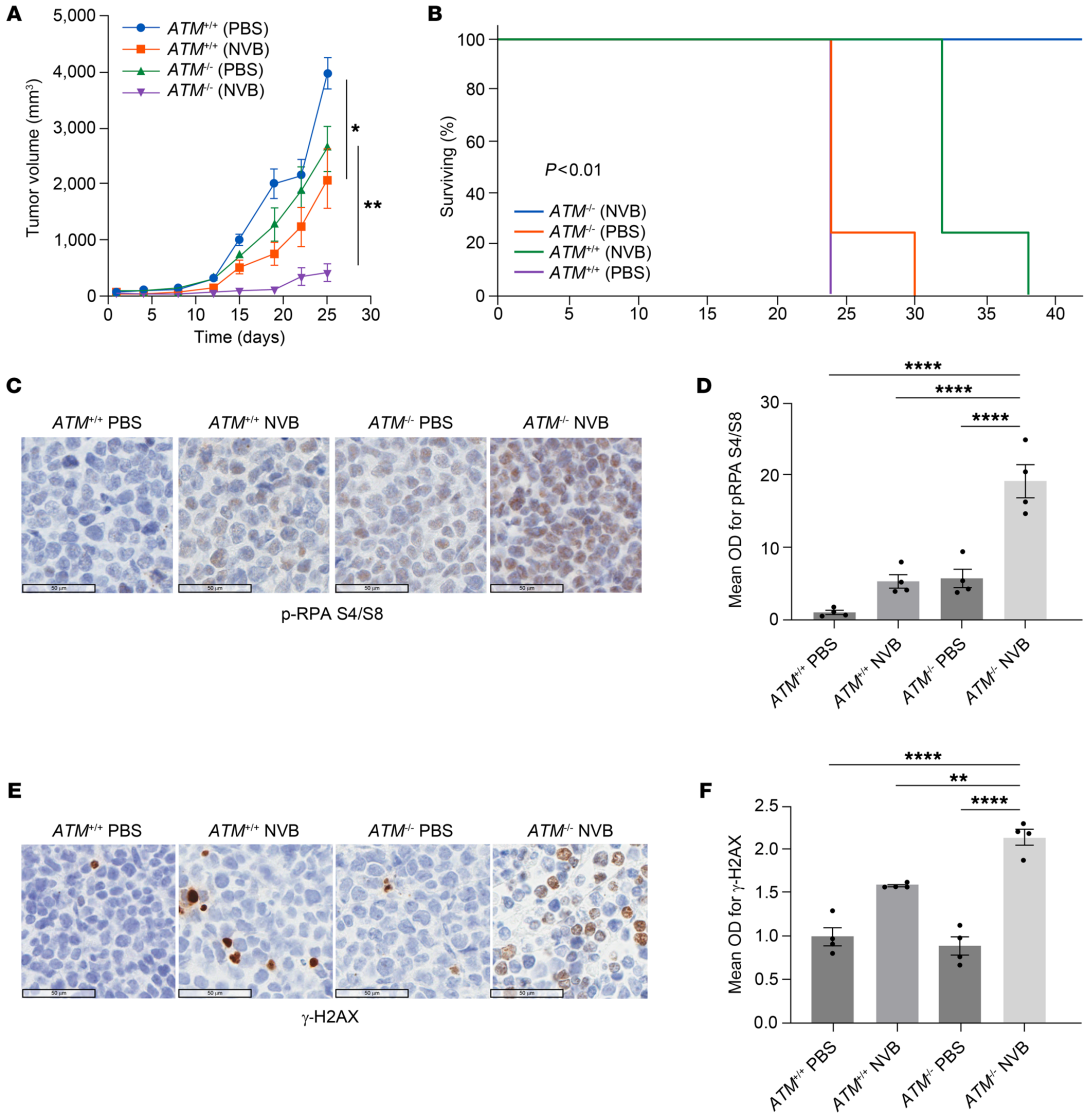
POLΘ depletion induces a significant antitumor effect in the in vivo MCL model, which is augmented with ATM deficiency. (**A**) Using immunodeficient mice, ATM-deficient and -proficient isogeneic Mino cells were engrafted and treated with NVB or vehicle (PBS). Each treatment group had 8 mice in total (4 females and 4 males; *P* value was calculated by 2-way mixed-model ANOVA). The antitumor effect was assessed through tumor measurements. (**B**) Overall survival of mice carrying MCL tumors with respective genotypes treated with NVB or vehicle (survival analysis using the Kaplan-Meier method was done using 4 mice per group). (**C** and **D**) Assessment of RS marker p-RPA S4/S8 via IHC in MCL tumor tissue following treatment with either NVB or vehicle (4 mice [1:1 male and female] per group; *P* value was calculated using 1-way ANOVA with Tukey’s post hoc test). (**E** and **F**) Assessment of DNA damage marker γ-H2AX in respective tumor types following treatment with either NVB or vehicle (4 mice [1:1 male and female] per group; *P* value was calculated using 1-way ANOVA with Tukey’s post hoc test). Scale bars: 50 μm. *P < 0.05; **P < 0.01; ****P < 0.0001. Data are shown as the mean ± SEM.

To examine the in vivo efficacy of POLΘ inhibition in MCL, immunodeficient mice were subcutaneously engrafted with isogeneic ATM-deficient (*ATM*^–/–^) and ATM-proficient (*ATM*^+/+^) Mino cells. Intraperitoneal NVB treatment commenced twice daily after tumor engraftment (100 mm^3^). Tumor growth was significantly reduced in ATM-proficient Mino xenografts treated with NVB compared with vehicle (Figure 7A). However, tumor growth inhibition was enhanced considerably in ATM-deficient Mino xenografts. No treatment-related morbidity or mortality was seen in the mice, and animal weights were similar in all groups (Supplemental Figure 6). Moreover, overall survival significantly increased in mice bearing ATM-proficient Mino xenografts treated with NVB (Figure 7B), which was further enhanced in ATM-deficient xenografts. Histopathological analyses showed a significant increase in RS marked by p-RPA32 S4/S8 (Figure 7, C and D) and unrepaired DNA damage evidenced by γ-H2AX (Figure 7, E and F) in ATM-proficient xenografts treated with NVB, and these biomarkers were further increased in NVB-treated ATM-deficient xenografts.

### POLΘ inhibition reduces the viability of primary MCL cells.

**Figure 8:**
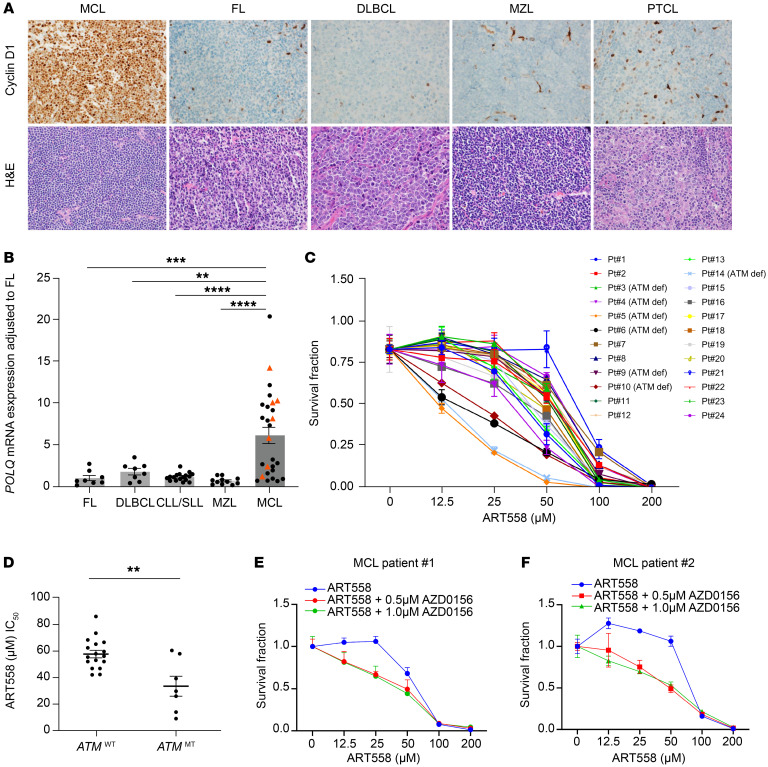
*POLQ* is overexpressed in MCL compared with other NHLs, and its inhibition showed an antitumor effect in primary tumor cells. (**A**) Cyclin D1 expression was assessed through IHC in MCL compared with other NHLs (×400 original magnification). (**B**) Assessment of *POLQ* expression in MCL compared with other types of NHL primary cells (*n* = 8 FL, *n* = 8 DLBCL, *n* = 17 CLL/SLL, *n* = 11 MZL, *n* = 27 MCL; *P* value was calculated by 1-way ANOVA with Tukey’s post hoc test). (**C** and **D**) POLΘ inhibition by ART558 causes a significant antitumor effect in primary MCL patient samples with increased antitumor effect seen in ATM-deficient compared with ATM-proficient primary MCL cells (experiments were done in triplicates, and *P* value was calculated by *t* test). (**E** and **F**) Concurrent inhibition of ATM and POLΘ increases the antitumor effect compared with POLΘ inhibition alone in primary MCL cells (experiments were done in triplicates). FL, follicular lymphoma; DLBCL, diffuse large B cell lymphoma; MZL, marginal zone lymphoma; PTCL, primary cutaneous T cell lymphoma; CLL/SLL, chronic lymphocytic leukemia/small lymphocytic lymphoma; Pt, patient. Data are shown as the mean ± SEM in **B** and **D** and the mean ± SD in **C**, **E**, and **F**. Red triangles in **B** represent ATM-deficient MCL primary samples. **P < 0.01; ***P < 0.001; ****P < 0.0001.

Having validated the effect of POLΘ inhibition in MCL using human cell lines, we next assessed the dependence on *POLQ* expression of primary tumor cells from patients with NHL. MCL exhibited the highest expression of cyclin D1 compared with most other NHL subtypes (Figure 8A and Supplemental Figure 7A). Consistent with our finding that cyclin D1 overexpression increases *POLQ* expression in cells, MCL primary cells exhibited the highest *POLQ* expression compared with other types of NHL, such as follicular lymphoma, chronic lymphocytic leukemia/small lymphocytic lymphoma, marginal zone lymphoma, and diffuse large B cell lymphoma, where cyclin D1 overexpression is not commonly observed (Figure 8B).

Subsequently, we tested the antitumor activity of POLΘ inhibition in primary MCL cells. Treatment of primary cells obtained from 24 MCL patients with ART558 showed significantly compromised viability (Figure 8C). Indeed, validating our cell line data, primary MCL cells with ATM deficiency were more affected by ART558 compared with ATM-proficient primary MCL cells (Figure 8D and Supplemental Figure 7B). As in the MCL cell lines, simultaneous inhibition of ATM and POLΘ showed enhanced antitumor activity in primary ATM-proficient MCL primary cells compared with POLΘ inhibition alone (Figure 8, E and F). In summary, these results in primary MCL cells confirm that inhibition of POLΘ is effective for *ATM*-mutated MCL and that concurrent inhibition of ATM and POLΘ is a promising therapeutic strategy for ATM-proficient MCL.

## Discussion

Our study demonstrates that overexpression of cyclin D1 increases RS, amplifying the reliance of cells on the MMEJ pathway for repairing DNA replication–associated damage, particularly during the mitotic phase of the cell cycle. This reliance intensifies with ATM deficiency. Moreover, cyclin D1 directly upregulates *POLQ* expression, which is further augmented by ATM deficiency. This heightened dependence on MMEJ may hold significance in cancers with high cyclin D1 expression, notably MCL, presenting a promising target for therapeutic intervention.

*CCND1* is one of the most amplified genes in human cancers, but its effects on RS and DNA damage repair have not been extensively characterized. Our study demonstrates that cyclin D1 overexpression induces RS in the context of MCL. Although *CCND1* is an oncogene known for its cell cycle regulatory function (18), recent studies have also identified the direct binding ability of cyclin D1 to gene enhancers, affecting respective gene transcription (52, 59). Here, we have demonstrated that cyclin D1 directly binds to the *POLQ* promoter and enhances *POLQ* transcription, indicating that MMEJ is likely required for the genomic integrity and survival of cyclin D1–overexpressing cells. The impact of these findings may extend beyond MCL, as cyclin D1 overexpression is seen in about 10%–20% of NHL and other hematologic malignancies (33); in solid tumors, including 50% of breast and colon cancers (60, 61); and in 80% of pancreatic cancer (62). Therefore, assessment of RS and MMEJ pathway dependence may be warranted in these other tumor types driven by oncogene expression.

Additionally, our results illustrate the synergistic interaction between POLΘ and ATM deficiencies in cells overexpressing cyclin D1, which is significantly stronger compared with cells with lower levels of cyclin D1 expression. Although previous studies in mice have indicated that concurrent deficiencies in POLΘ and ATM can be partially synthetic lethal (63), our data suggest that cyclin D1 overexpression may be a useful biomarker for combined POLΘ and ATM inhibition in cancer treatment. Furthermore, unlike in MCL, inhibiting POLΘ in ATM-deficient models that are BRCA proficient without elevated cyclin D1 expression demonstrated only a minimal additive effect (64). Our data support these findings, showing that ATM depletion in cells that do not overexpress cyclin D1 had minimal impact on RS and MMEJ dependence.

Notably, a synergistic antitumor effect from the simultaneous inhibition of ATM and POLΘ was observed only in settings with cyclin D1 overexpression in an HR repair proficient background. This mechanistic insight highlights a distinct disruption of DNA replication and variations in DSB repair pathway dependency in ATM-deficient, HR-proficient cells with cyclin D1 overexpression. Consequently, our findings may have broader implications for other HR-proficient cancers exhibiting concurrent cyclin D1 overexpression and ATM deficiency and suggest a biomarker-driven approach for the development of inhibitors of POLΘ.

Previous studies have demonstrated that POLΘ is essential for sealing postreplicative single-strand DNA gaps in BRCA-deficient cancer cells (64, 65). Additionally, the function of POLΘ has been linked to maintaining genomic stability in HR-deficient solid tumor malignancies (64). To our knowledge, no studies have examined the role of POLΘ in maintaining chromosomal stability in hematologic malignancies characterized by cyclin D1 overexpression, particularly in HR-proficient backgrounds. Our results indicate that POLΘ is crucial for reducing DNA RS, especially in HR-proficient cells subjected to perturbations in oncogenes and tumor suppressor genes, including *CCND1* and *ATM* respectively. Moreover, highly proliferative MCLs are highly dependent on MMEJ-mediated DSB repair during the mitotic phase of the cell cycle, consistent with previous studies that demonstrated the activation of POLΘ by PLK1 and the role of MMEJ in repair of damaged DNA during mitosis (5, 6). However, considering the role of POLΘ in sealing single-strand DNA gaps predominantly during the S phase of the cell cycle (64, 65), it is important to note that its functions are not restricted to mitosis. POLΘ also should be active in the S phase, even in cells with high HR proficiency. These findings are significant to the field of malignant hematology, where, despite the high proliferative index of cancer cells, the biology of RS and mechanisms of DNA damage repair remain understudied (66).

Our study has limitations, particularly related to the exact mechanism for the increased MMEJ dependence resulting from ATM depletion in cyclin D1–overexpressing cells. Although we found that ATM deficiency further increased *POLQ* transcription in cyclin D1 overexpressed cells, the mechanism behind this observation needs to be elucidated. Previous studies have shown that ATM suppresses MMEJ by regulating DNA end degradation and controlling the activity of the Mre11 nuclease, which is essential for initiating MMEJ (54). Consequently, ATM deficiency leads to increased DNA end resection, promoting the use of MMEJ for DSB repair (54).

It is unlikely that the increased POLΘ protein expression we observed with cyclin D1 overexpression — an effect further heightened by ATM deficiency — is merely a consequence of cell cycle effects related to cyclin D1 overexpression or ATM deficiency. In our experimental system, neither cyclin D1 overexpression nor ATM deficiency significantly altered the cell cycle dynamics of the malignant cells. Although its function is most critical during mitosis, POLΘ protein levels are relatively lower in the mitotic phase compared with other cell cycle stages (5, 6). Importantly, ATM deficiency does not induce cell cycle arrest, while cyclin D1 overexpression is expected to enhance cell cycle progression from the G_1_ to the S phase. Consequently, our findings of increased *POLQ* expression in the context of cyclin D1 overexpression and ATM deficiency appear to be a direct result of these genetic alterations rather than a secondary effect stemming from cell cycle perturbations.

In conclusion, our work elucidates the impact of cyclin D1 overexpression on RS, particularly emphasizing its implications for MCL. We identified POLΘ as a critical mediator in the cellular management of RS induced by cyclin D1 overexpression, consequently decreasing the prevalence of single-stranded DNA and increasing cellular dependency on MMEJ-mediated DSB repair, particularly during the mitotic phase. This process is essential for maintaining cell proliferation and viability. Moreover, the concurrent reduction of ATM in the context of cyclin D1 overexpression, even in an HR-proficient background, exacerbates RS, leading to enhanced POLΘ expression and even greater reliance on MMEJ. These preclinical results demonstrate the therapeutic potential of targeting POLΘ in oncogene-driven hematologic cancers, especially in MCL, where cyclin D1 overexpression is ubiquitous and ATM deficiency is common, but may extend to other oncogene-driven hematologic cancers. Further, these preclinical data strongly support bringing our findings from the bench to the bedside for relapsed and/or refractory MCL targeting the MMEJ pathway through POLΘ inhibition with or without ATM inhibition.

## Methods

### Sex as a biological variable.

We included both male and female animals in our study, and the findings were consistent across both sexes.

### Cell culture.

U2OS (ATCC; HTB-96), HEK293T (ATCC; CRL-3216), UPN2 (67), and Granta-519 (DSMZ; ACC 342) cells were cultured in DMEM (Gibco). RPE cells (ATCC; CRL-4000) were cultured in DMEM/F-12 (Gibco). Jeko (ATCC; CRL-3006), Mino (ATCC; CRL-3000), JVM-2 (ATCC; CRL-3002), Rec1 (ATCC; CRL-3004), and MCIR cells (68) were cultured in RPMI-1640 medium (Gibco). Z138 cells (ATCC; CRL-3001) were cultured in IMDM (Gibco). All media were supplemented with 10% FBS (Sigma-Aldrich) and 1% penicillin/streptomycin (Life Technologies). Cells were maintained at 37°C in a humidified incubator with 5% CO_2_.

### Lentivirus-mediated gene manipulation.

For overexpressing CCND1 in cells, 2xHA-tagged CCND1 was integrated into a pLV-EF1a-IRES-Blast vector (Addgene; 85133), and cells were lentivirally transduced with control or CCND1. After blastidicin selection for 96 h, cells were used to perform experiments.

For CRISPR-mediated gene knockout, cells were lentivirally transduced with Cas9 and a sgRNA simultaneously using a lentiCRISPR v2 vector (Addgene; 52961). After puromycin selection for 72 h, cells were used to perform experiments.

For CRISPRi-mediated gene knockdowns, cells were transduced with dCas9-KRAB using the lentivirus generated by pHR-SFFV-KRAB-dCas9-P2A-mCherry (Addgene; 60954). mCherry-positive cells were sorted using a FACSAria II sorter (BD Biosciences) and subsequently transduced with a control (nontargeting [NT]) or sgRNA targeting CCND1 using the lentivirus generated by lentiGuide-Puro vector (Addgene; 52963). After puromycin selection for 72 h, cells were used to perform experiments.

For competitive assay, Cas9-expressing cells were lentivirally transduced with a control or sgRNA targeting *POLQ* in a pLKO5.sgRNA.EFS.GFP vector (Addgene; 57822) with or without a control or sgRNA targeting *ATM* in a pLKO5.sgRNA.EFS.tRFP657 vector (Addgene; 57824).

Lentivirus was produced by transient transfection of HEK293T cells with viral plasmids along with gag-expressing, pol-expressing (psPAX2; Addgene; 12260), and env-expressing (pMD2.G; Addgene; 12259) plasmids using the calcium-phosphate method (Takara Bio).

Sequences of the sgRNA used for CRISPR-mediated gene knockout or gene silencing in this study are provided in Supplemental Table 1.

### Generation of CRISPR-mediated knockout and knockin cells.

For generating *ATM* or *POLQ* knockout cells, a ribonucleoprotein (RNP) complex was formed by Alt-R S.p. HiFi Cas9 and CRISPR-Cas9 sgRNA targeting *ATM* or *POLQ* (Integrated DNA Technologies). MCL cells were resuspended in SF Nucleofector Solution with supplement (Lonza) and then mixed with the Cas9/sgRNA RNP complex and Alt-R Cas9 Electroporation Enhancer (Integrated DNA Technologies). The Cas9/sgRNA RNP complex was delivered to the cells using the 4D-Nucleofector system (Lonza).

For generating endogenously 3xFLAG-tagged *POLQ* Jeko or V5-tagged *POLQ* U2OS knockin cells, the Cas9/sgRNA RNP complex together with a template was delivered. Jeko and U2OS cells were resuspended in SF or SE Nucleofector Solution with supplement (Lonza), respectively. The double-stranded DNA template consisted of 500 bp upstream and 500 bp downstream of the cutting site by Cas9 together with a 3xFLAG or V5 tag. Of note, the codon usage of *POLQ* residue 6R of the template DNA was changed from CGG to CGT to destroy the PAM sequence, which prevented the inserted template from being cut by Cas9 after knockin. After electroporation, the cells were incubated for 24 h with an homology-directed repair (HDR) enhancer (Integrated DNA Technologies) to increase knockin efficiency. Media was changed the following day, and 2 days later cells were seeded in a 96-well plate to isolate single clones. Individual clones were tested for genomic editing analyses using immunoblotting and genomic PCR with subsequent Sanger sequencing. The sgRNAs used for gene knockout and knockin are listed in Supplemental Table 1.

### Viral transduction.

Retroviruses for human cells and lentiviruses were produced by transient transfection of 293T cells with viral plasmids along with gag-, pol-, and env-expressing plasmids using the calcium-phosphate method (69). Retrovirus transduction to the cells was performed using Retronectin (Takara Bio Inc.).

### Western blot analysis.

Primary antibodies used were anti-CCND1 (rabbit polyclonal; CST; catalog 2922S), anti-ATM (rabbit monoclonal; CST; catalog 2873S; clone D2E2), anti–γ-H2AX (rabbit monoclonal; CST; catalog 9718S; clone 20E3), anti-RPA (rabbit polyclonal; CST; catalog 52448), anti–p-RPA S8 (rabbit monoclonal; CST; catalog 54762; clone D6X3V), anti–cleaved-PARP (rabbit monoclonal; CST; catalog 5625T; clone D64E10), anti–cyclin-B1 (rabbit polyclonal; CST; catalog 4138S), anti-CHK1 (mouse monoclonal; CST; catalog 2360S; clone 2G1D5), anti-pCHK1(rabbit monoclonal; CST; catalog 2348; clone 133D3), anti-KAP1 (mouse monoclonal; Abcam; catalog ab22553; clone 20C1), anti-pKAP1 (rabbit polyclonal; Abcam; catalog ab70369), anti-V5 (rabbit monoclonal; CST; catalog 13202; clone D3H8Q), anti-FLAG (mouse monoclonal; Sigma-Aldrich; catalog F1804; clone M2), anti–α-tubulin (rabbit monoclonal; CST; catalog 2125; clone 11H10), anti–β-actin (rabbit monoclonal; CST; catalog 4970; clone 13E5), anti-vinculin (rabbit monoclonal; CST; catalog 13901; clone E1E9V), and anti-GAPDH (rabbit monoclonal; CST; catalog 5174; clone D16H11).

### Reverse transcription PCR.

Total RNA was extracted using an RNeasy Mini Kit (Qiagen). cDNA synthesis was performed using the SuperScript IV First-Strand cDNA Synthesis kit (Thermo Fisher Scientific). qPCR was performed using Power SYBR Green PCR Master Mix (Thermo Fisher Scientific) and the Quant Studio 7 Flex Real-Time PCR System (Thermo Fisher Scientific). ΔCt was calculated using *GAPDH* as a control and normalized to control cell lines if not otherwise specified in a figure legend. Reverse transcription qPCR (RT-qPCR) assays were performed in technical triplicate. Sequences of the primers used for RT-qPCR are as follows: hCCND1-qF1, TCTACACCGACAACTCCATCCG; hCCND1-qR1, TCTGGCATTTTGGAGAGGAAGTG; hPolQ-qF1, CTTGTGGCATCTCCTTGGAGCA; hPolQ-qR1, AATCCCTTGGCTGGTCTCCATC; hATM-qF1, TGTTCCAGGACACGAAGGGAGA; and hATM-qR1, CAGGGTTCTCAGCACTATGGGA.

### DNA repair template assay.

U2OS cells carrying a DNA repair template reporter (DR-GFP, EJ5-GFP, and MMEJ-GFP) were lentivirally transduced with Cas9 and sgRNA (coexpressing puromycin resistance gene) with or without a control vector or CCND1 cDNA (coexpressing blasticidin resistance gene). After puromycin and blasticidin selection, 40,000 DNA repair template reporter cells were seeded in 12-well plates and adenovirally transduced with Isce-I the following day. Forty-eight hours after Isce-I transduction, GFP signals were analyzed by the CytoFLEX platform (Beckman Coulter). The signals were normalized to control cells. DR-GFP and EJ5-GFP cells were gifts from Jeremy Stark (Beckman Research Institute of the City of Hope, Duarte, California, USA). MMEJ-GFP cells were generated by lentivirally transducing a cassette into U2OS cells using a pLV-EF1a-IRES-puro vector (Addgene; 85132) as previously described (42).

### Competitive assay.

MCL cell lines were lentivirally transduced with Cas9 (coexpressing mCherry), and mCherry-positive cells were sorted by a FACSAria II sorter. These mCherry-positive cells were subsequently lentivirally transduced with a sgRNA targeting control or *POLQ* (coexpressing GFP) together with a sgRNA targeting control or *ATM* (coexpressing tRFP657), and changes in the frequency of GFP/tRFP657 double-positive cells were monitored. GFP and tRFP657 signals were analyzed by CytoFLEX (Beckman Coulter) every 3–4 days. The signals were normalized to the frequency of GFP-positive or GFP/tRFP657 double-positive cells at day 3.

### Cell survival assays.

For the clonogenic assay, 500 to 4,000 cells were seeded into 6-well plates, with the exact number adjusted based on the growth rate of each cell line. The following day, cells were treated as indicated. After 7–14 days, colonies were washed with PBS, fixed with methanol/acetic acid (3:1) fixation solution for 1 h, and stained with 0.5% crystal violet (prepared in 10% methanol) for 1 h. Stained plates were then imaged and analyzed using ImageJ (version 1.54; NIH).

For the CellTiter-Glo assay (Promega; catalog G7570), cells were plated in 96-well plates and treated as indicated the following day. After 5–7 days, CellTiter-Glo reagent (Promega; catalog G7573) was added according to the manufacturer’s protocol to assess cell viability. Luminescence was measured using a plate reader.

### Chromosomal breakage assay.

Jeko control or sg*POLQ* cells were exposed to 5 ng/mL MMC for 48 h. Cells were treated with 100 ng/mL of colcemid for 2 h, followed by a hypotonic solution (0.075 M KCl) for 20 minutes and fixed with 3:1 methanol/acetic acid. Slides were stained with Wright’s stain, and 50 metaphase spreads were scored for aberrations.

### Immunofluorescence.

After specific treatments or in the absence of treatment, cells were fixed with 4% paraformaldehyde for 15 minutes at room temperature. Cells were then permeabilized with 0.3% Triton X-100 for 10 minutes on ice, followed by blocking with 3% nonfat milk for 1 h at room temperature. The slides were stained with primary antibodies at 4°C overnight. Afterward, they were stained with secondary fluorescent-conjugated antibodies for 1 h at room temperature. The slides were scanned using a fluorescence microscope. At least 100 cells were counted for each sample. Foci quantification was performed using CellProfiler version 4.2.6 software (Broad Institute of MIT and Harvard).

Primary antibodies used were anti–γ-H2AX (mouse monoclonal; MilliporeSigma; catalog 05-636-MI; clone JBW301), anti–p-RPA2 S33 (rabbit polyclonal; Novus Biologicals; catalog NB100544), and anti-V5 (rabbit monoclonal; CST; catalog 13202; clone D3H8Q).

### Comet assay.

The alkaline comet assays were performed to detect both single- and double-stranded DNA breaks. One thousand cells, suspended in PBS, were mixed with 50 μL low melting point agarose and pipetted onto comet slides (R&D Systems; catalog 4250-050-K). Once the agarose solidified, the slides were immersed in a lysis solution for 18 h to facilitate cell lysis. Following lysis, the slides were incubated in an alkaline unwinding solution for 1 h to denature the DNA. Electrophoresis was performed at 21 V for 45 minutes in an alkaline electrophoresis solution. The slides were stained with SYBR green solution, scanned by a fluorescence microscope, and analyzed using CometScore software (version 2.0) (TriTek Solutions).

### DNA fiber assays with S1 nuclease.

For DNA fiber assays, cells (sgNT and sg*POLQ* Jeko cells) were seeded at 50% confluence in 6-well plates 1 day before the experiment. The next day, they were sequentially incubated with CldU (100 μmol/L, 30 minutes) followed by IdU (100 μmol/L, 2 h) at 37°C. After each incubation step, cells were washed 3 times with PBS and permeabilized using CSK buffer (0.5% Triton X-100, 10 mmol/L HEPES, 300 mmol/L sucrose, 100 mmol/L NaCl, and 3 mmol/L MgCl_2_) for 10 minutes at room temperature. Following a wash with S1 nuclease buffer (50 mmol/L NaCl, 300 mmol/L sodium acetate pH 4.6, 10 mmol/L zinc acetate, and 5% glycerol), cells were incubated at 37°C for 30 minutes in the presence or absence of S1 nuclease (20 U/mL). Nuclei were then washed with PBS, resuspended in PBS with 0.1% BSA, harvested using a cell lifter, pelleted, and mixed with melted agarose to form plugs by incubating at 4°C for 45 minutes. These plugs were then digested overnight at 50°C in proteinase K solution before being washed 4 times with buffer and incubated in combing buffer overnight at 4°C. DNA fibers were stretched onto coverslips (COV002-RUO; Genomic Vision) using the FiberComb Molecular Combing System (MCS001) and processed for immunostaining. Coverslips were incubated overnight at 37°C with rat anti-BrdU (Abcam; ab6326; clone BU1/75 [ICR1]) and mouse anti-BrdU (BD Biosciences; 347580; clone B44), both diluted in BlockAid (Invitrogen; B10710), followed by goat anti-rat Cy5 (Abcam; ab6565; polyclonal) and goat anti-mouse Cy3 (Abcam; 97035; polyclonal) for 45 minutes at 37°C. To detect single-stranded DNA, samples were further incubated with mouse anti-ssDNA antibody (Developmental Studies Hybridoma Bank autoanti-ssdna) for 1 h and 15 minutes at 37°C, followed by goat anti-mouse BV480 (Jackson ImmunoResearch; 115-685-166; polyclonal) for 45 minutes at 37°C. After immunostaining, coverslips were air-dried, mounted, and scanned using a FiberVision scanner (Genomic Vision).

### Cell cycle synchronization and cell cycle analysis.

Cells were synchronized at the G1/S boundary using a double thymidine block. Cells were first treated with 2 mM thymidine for 16 h, followed by release into fresh thymidine-free medium for 8–10 h to allow progression through the cell cycle. Cells were then subjected to a second 2 mM thymidine treatment for another 16 h, ensuring a more uniform synchronization at the G1/S transition. Mitotic synchronization was achieved using nocodazole (100 ng/mL, 12 h) or RO3306 (8 μM, 18 h) followed by a 1 h release in fresh medium. Cells were fixed in 70% ethanol, stained with propidium iodide, and analyzed by flow cytometry to determine cell cycle distribution.

### Luciferase assay.

HEK293T cells were seeded in 12-well culture plates at a density of a 100,000 per well. At 16 h after seeding, the cells were transfected with pGL4.1 *POLQ* promoter (coexpressing Firefly luciferase), pLV-IRES-Blast EV or pLV-IRES-Blast CCND1, and pGL4.71 vector (coexpressing Renilla luciferase) using Lipefectamine LTX Reagent with PLUS Reagent (Thermo Fisher Scientific). The cells were harvested 48 h after transfection and assayed for the luciferase activity by means of the luciferase assay system (Promega) and a luminometer (FLUOstar OPTIMA; BMG LABTECH). Promoter activity was calculated as the ratio of Renilla to Firefly luciferase.

### ChIP-qPCR assay.

CRISPRi-mediated *CCND1* knocked down Jeko cells were lentivirally transduced with an EV or 2xHA-Thirty-million Jeko control or *CCND1* knockdown cells. ChIP was performed using a SimpleChIP kit (Cell Signaling Technology; 9002) with an antibody against HA (Abcam; catalog ab9110; polyclonal) following the manufacturer’s recommendations. Purified DNA was then subjected to RT-qPCR using SYBR Select Master Mix (Applied Biosystems). Sequences of the primers used for ChIP-qPCR are as follows: *POLQ* #1-F, GAGCTACTTCCCTGATCTACCT; *POLQ* #1-R, CCATACTGACCTAAAAGCCTTCC; *POLQ* #2-F, AGCATGGCCTTCCTATTCAAAC; *POLQ* #2-R, CTAAGACTTCCGGCCTCCAA; *POLQ* #3-F, TTGGAGGCCGGAAGTCTTAG; *POLQ* #3-R, ATCTTCCCGCCAGTCTTCAA; *POLQ* #4-F, CGAGTCTATGGCTTTCGGGT; *POLQ* #4-R, TTCCCGCCAGTCTTCAAACT; ATM #1F, AATCGCTTCCGCCTAGAGAAAG; ATM #1R, CTCTCACCCACCCTCTTCGC; ATM #2F, GTCGTCACCTTCGTCCGCAG; and ATM #2R, GCCTGCGCCATGTCCAC.

### Animal experiments.

One million *ATM*-WT or *ATM*-knockout Mino cells were subcutaneously injected into 7-week-old male and female NOD.Cg-*Prkdc*^scid^ Il2rg^tm1Wjl^/SzJ mice, purchased from The Jackson Laboratory. Male and female mice were used in a 1:1 ratio, and each treatment group had 8 mice (4 males and 4 females). After confirming tumor engraftment, mice were treated with NVB (75 mg/kg) or PBS twice a day via i.p. injection for 3–4 weeks. Tumors were measured every 2 to 3 days using an electronic caliper, and tumor volumes were calculated using the formula L × W × W/2. For IHC analysis, tumors were excised and fixed with formaldehyde. Mice with a tumor of more than 20 mm in length or width were euthanized.

### Primary patient sample analysis.

Primary patient samples were obtained from the Mayo Clinic Lymphoma and Predolin Foundation Biobank. Primary cells (20,000 per well) were plated in 96-well plates in triplicates, and a CellTiter-Glo assay was conducted. Immunoblotting was conducted on selected patient samples where sufficient cells were obtained to assess for ATM deficiency (rabbit monoclonal; CST; catalog 2873S).

### IHC.

IHC was performed on the Leica Bond III automated staining platform using the Leica Biosystems Refine Detection Kit (DS9800). FFPE tissue sections were baked for 30 minutes at 60°C and deparaffinized (Leica AR9222) prior to staining. Primary antibodies were incubated for 30 minutes, visualized via DAB, and counterstained with hematoxylin (Leica DS9800). The samples were rehydrated in graded alcohol and placed on coverslips with HistoreCore Spectra CV mounting medium (Leica 3801733).

Antibodies used were as follows: (a) p-RPA32 (S4/S8) (Bethyl Laboratories; catalog A300-245A; polyclonal) was run at 1:2,000 concentration with a 30 M citrate antigen retrieval (Leica ER1 AR9961). (b) Phospho-Histone H2A.X (S139) (MilliporeSigma; catalog 05-636; clone JBW301) was run at 1:40,000 dilution with a 20 M EDTA antigen retrieval (Leica ER2 AR9640).

### Statistics.

Data were analyzed and visualized using GraphPad Prism (version 10.2.2; GraphPad Software, LLC). Statistical comparisons were made using either a 2-tailed Student’s *t* test or a Mann-Whitney *U* test for 2-group analyses. For multigroup comparisons, 1-way ANOVA followed by Tukey’s or a 2-way mixed-model ANOVA was performed. Drug synergy was calculated using the Bliss Independence Model in which a Bliss synergy score of more than 5 was considered synergistic (70, 71). Statistical significance was defined as *P* < 0.05.

### Study approval.

The study was approved by the IRBs and IACUCs at the Dana-Farber Cancer Institute and Mayo Clinic Minnesota. All procedures were conducted in accordance with the Declaration of Helsinki.

### Data availability.

All data utilized for analysis are provided in the Supporting Data Values file. Requests for raw or analyzed data and materials related to this article that are not included in the manuscript within main text or supplemental materials will be reviewed by the respective institutions to determine if there are any intellectual property or confidentiality restrictions. Any shareable data or materials will be provided through a material transfer agreement.

## Author contributions

JPA, SA, GZ, ZL, GIS, TW, JRC, and AD designed the studies. JPA, SA, GZ, YH, LM, DI, SM, KP, GZ, LJ, DY, MM, KG, and XW conducted the studies. JPA, SA, GZ, and AD wrote the manuscript, and GIS and TW critically appraised it. JPA led the study with significant contributions from SA and GZ, which defined the order of co–first authors. RLK was the pathologist who read the IHC images. All authors reviewed and approved the final manuscript.

## Supplementary Material

Supplemental data

Unedited blot and gel images

Supporting data values

## Version 1 07/03/2025

In-Press Preview

## Version 2 09/02/2025

Electronic publication
