# Supplementary material for: Cyclin D1 overexpression induces replication stress and microhomology-mediated end-joining dependence in mantle cell lymphoma
Source: J Clin Invest. 2025 Jul 3;135(17):e193006. doi: 10.1172/JCI193006 (PMC12404763; doi:10.1172/JCI193006)
Supplement: Supplemental data [file jci-135-193006-s206.pdf]

Supplemental Figure 1: (A) Assessment of cell proliferation with cyclin D1 overexpression in U2OS (done in triplicates). **(B)** Immunoblot depicting cyclin D1 overexpression in reporter cells used to assess DNA double-strand break repair pathways with respective genetic alterations (Figure 1D). **(C-E)** valuation of V5 tagged to N terminus of endogenous POLΘ in U2OS cell line. EV: empty vector; OE: overexpressed; sg: single guide RNA; ****p <0.0001. Error bars represent SEM.

Supplemental Figure 1

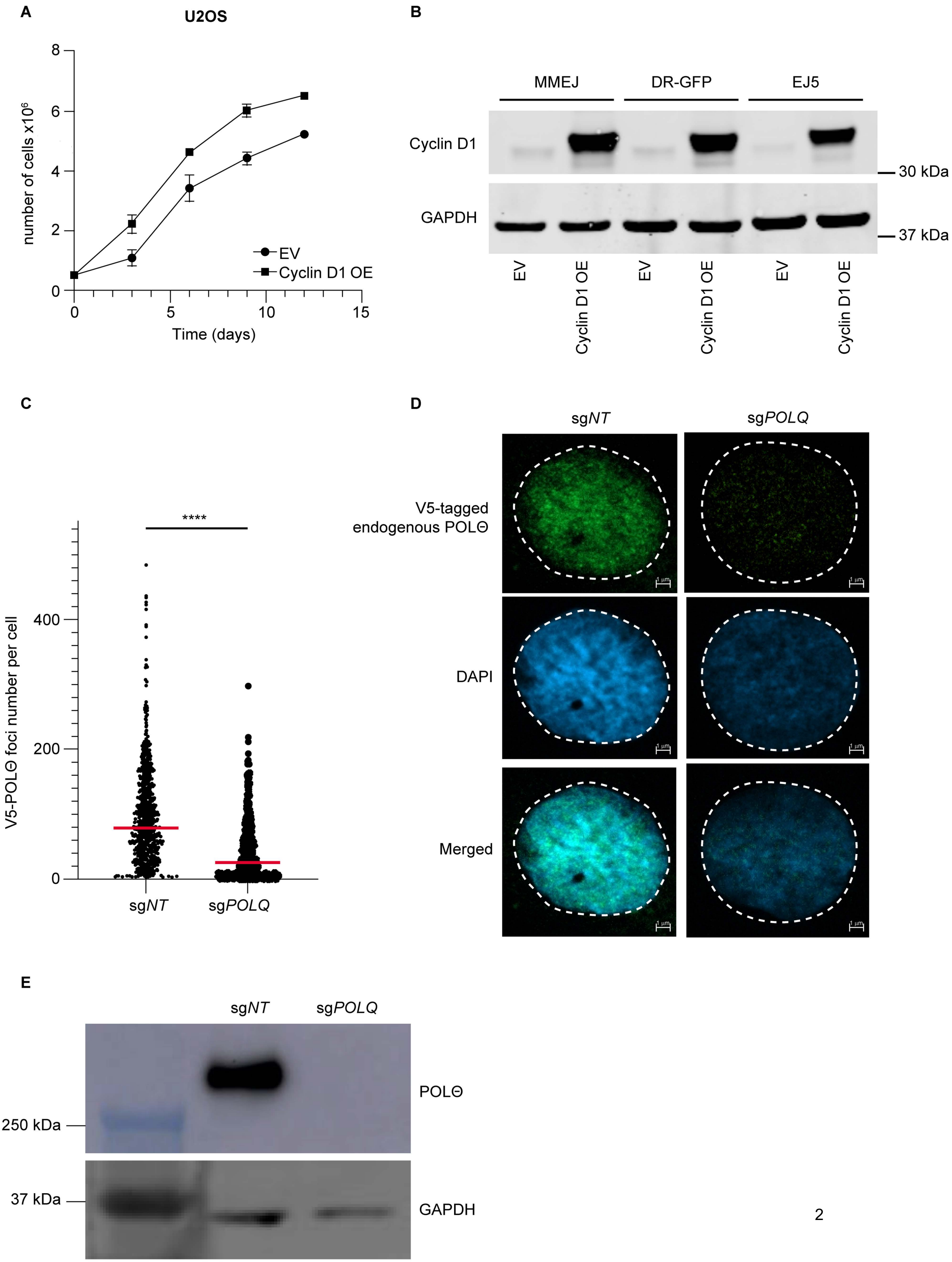

Supplemental Figure 2: (A) Immunoblot depicting POLΘ in POLΘ depleted isogeneic Jeko cells. **(B)** Cell cycle analysis using propidium iodide to assess G_1, S and M-phase arrest on Jeko cells. **(C)** Cell cycle analysis using propidium iodide to assess M-phase arrest. **(D** and **E)** Antitumor effect of ATR and PARP inhibition in POLΘ–proficient and deficient backgrounds in Jeko cells (done in triplicates). sg: single-guide; NT: non-target. Error bars represent SEM.

Supplemental Figure 2

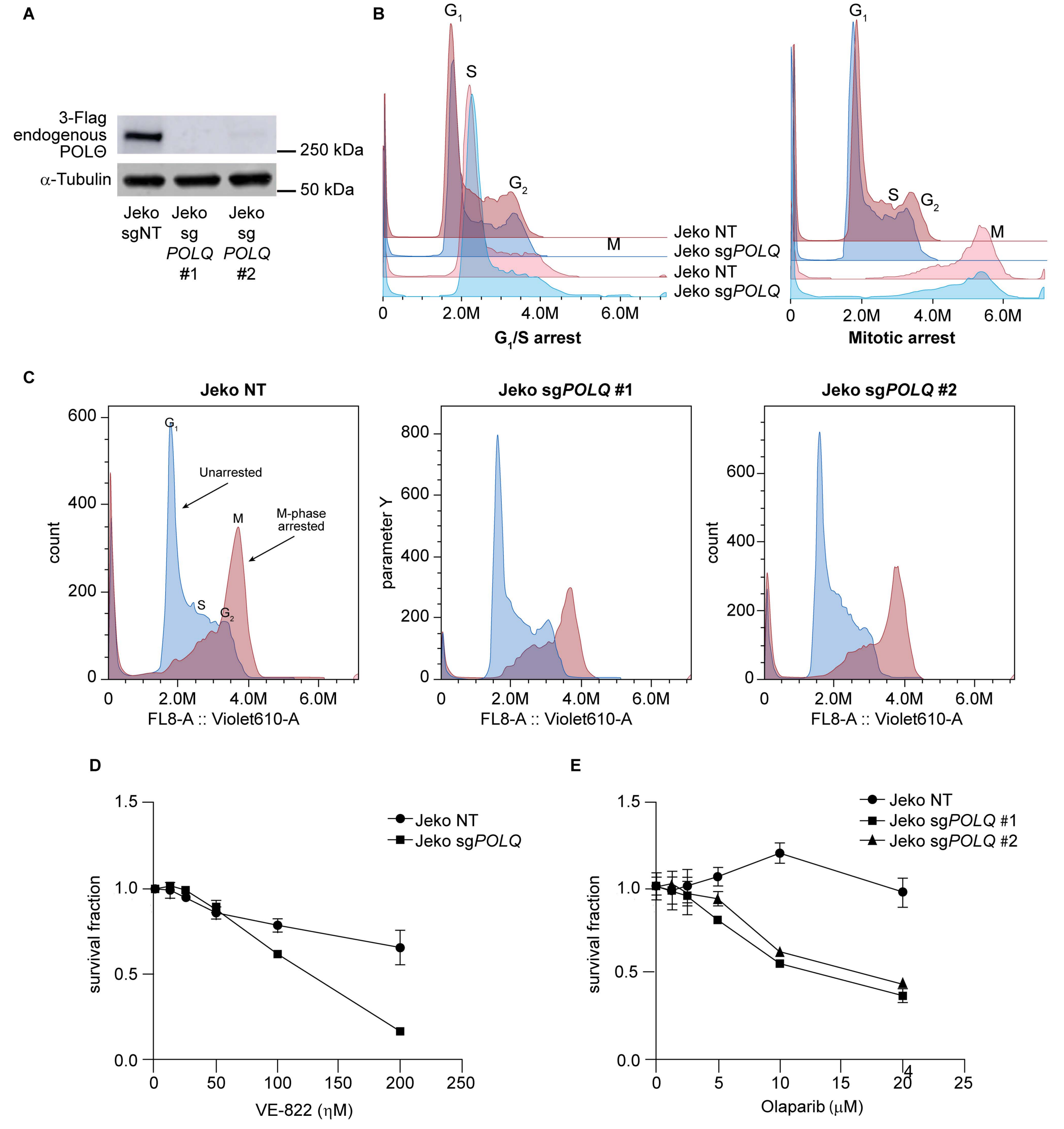

Supplemental Figure 3: (A) Cell cycle assessment with CRISPRi-mediated decreased expression of cyclin D1 in Jeko cells. **(B)** Immunoblot depicting Jeko cells used for Figure 3F in which CRISPRi induced decreased expression of endogenous cyclin D1 and overexpression of HA-tagged ectopic cyclin D1. **(C)** Immunoblot assessing HA-tagged ectopic cyclin D1 in U2OS cells used for experiments in supplemental Figure 3G. **(D** and **E)** Assessment of *ATM* mRNA through PCR in U2OS cells with and without cyclin D1 overexpression (D, done in sextuplicate) and in Jeko cells with and without decreased expression of cyclin D1 (E, done in quintuplicate). **(F)** Assessment of the enrichment of *ATM* promoter region through chromatin immunoprecipitation in HA-tagged cyclin D1 overexpressed Jeko cells with CRISPRi mediated decreased expression of endogenous cyclin D1 (done in quadruplicates). **(G)** Evaluating cyclin D1 binding to *POLQ* promoter region through chromatin immunoprecipitation in HA-tagged cyclin D1 overexpressed U2OS cells (done in triplicates). OE: overexpressed; sg: single guide; NT: non-target; EV: empty vector; ns: not significant. CRISPRi: CRISPR interference. Error bars represent SEM.

Supplemental Figure 3

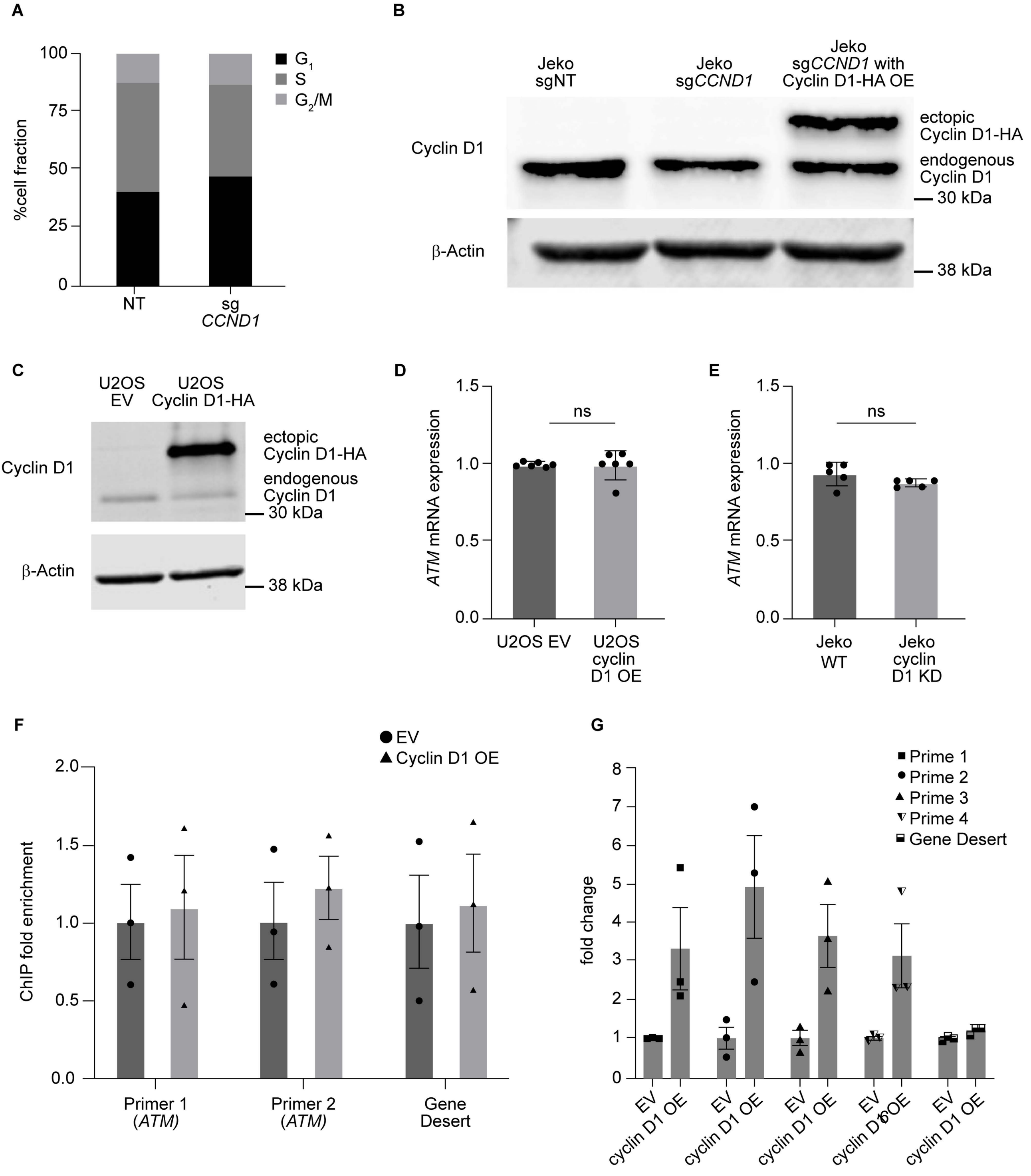

Supplemental Figure 4: (A-C) Immunoblot depicting cyclin D1 overexpression and ATM deficiency in reporter cells used to assess DNA double-strand break repair pathways (Figure 4E and Supplemental Figure 4, I and J). **(D)** Immunoblot assessment of ATM in isogeneic ATM deficient and proficient cells. **(E)** Assessment of *POLQ* expression in cyclin D1 overexpressing U2OS cells with and without ATM deficiency (done in triplicates). **(F)** Assessment of *POLQ* expression in primary MCL cells**. (G** and **H)** Assessment of *POLQ* (H) and CCND1 (I) expression in MCL primary cells with and without ATM mutation using publicly available gene expression dataset (ref 56). **(I** and **J)** Assessment of NHEJ **(I)** and HR **(J)** activity with cyclin D1 overexpression and ATM deficiency using standardized reporter assays (done in quadruplicates). OE: overexpressed; KO: knock-out; NHEJ: non-homologous end-joining; EV: empty vector; wt: wild-type; mut: mutant; MCL: mantle cell lymphoma; ns: not significant; **p<0.01. Error bars represent SEM.

Supplemental Figure 4

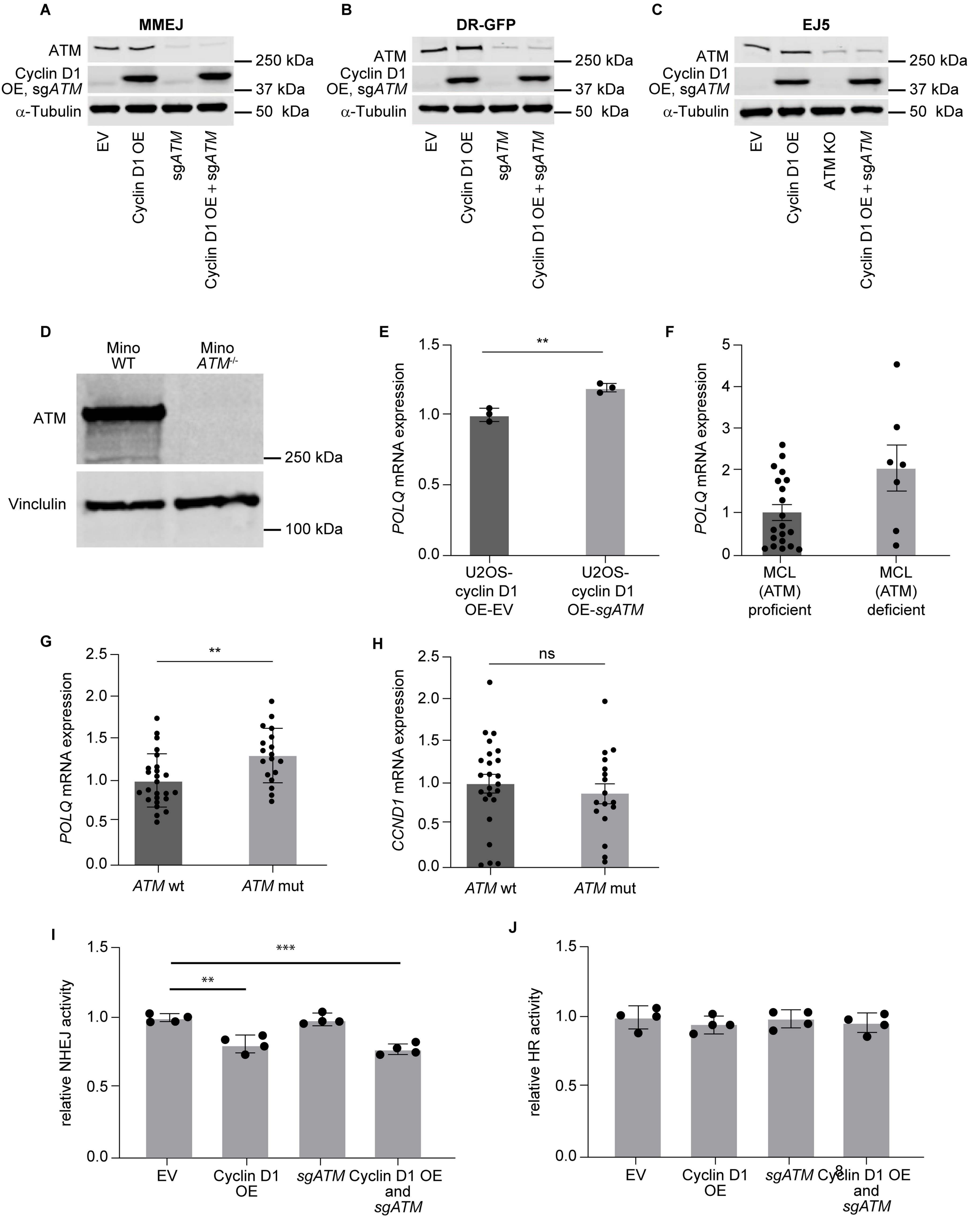

Supplemental Figure 5: (A) Assessment of antiproliferative effect with *POLQ* depletion in multiple MCL cell lines (done in triplicates). **(B)** Probing for ATM pathway activity after radiation (2 Gy) in multiple MCL cell lines in which Granta, UPN2, and MCIR were identified as ATM-deficient. **(C** and **D)** Assessment of sensitivity to POLΘ inhibition using ART558 **(C)** and NVB **(D)** in U2OS cells with respective genetic backgrounds (cyclin D1 overexpressed and ATM-deficient, done in triplicates). sg: single guide; NT: non target; OE: overexpressed; GFP: green fluorescent protein; NVB: novobiocin; Gy: Gray. Error bars represent SEM.

Supplemental Figure 5

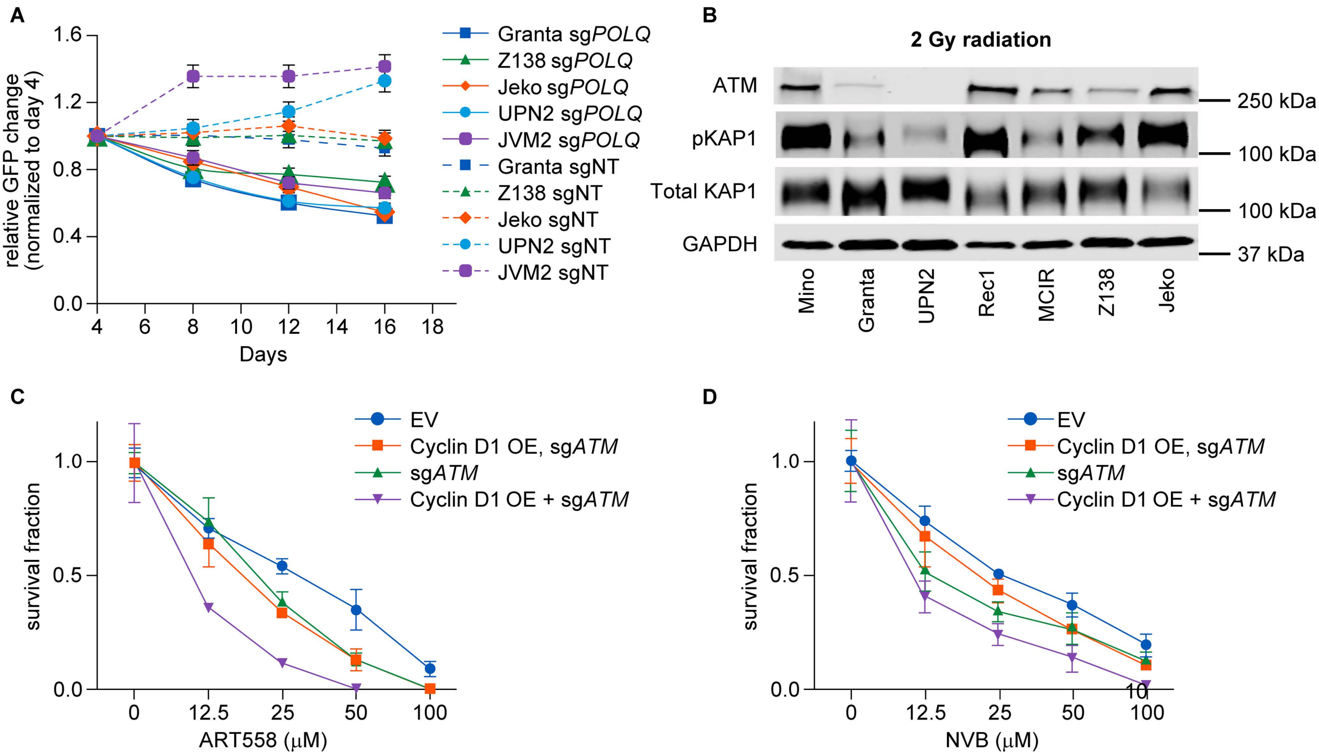

Supplemental Figure 6: Measurement of body weight of mice bearing respective tumor types along the treatment period. PBS: phosphate buffered saline; NVB: novobiocin. Error bars represent SEM. Each data point represents the mean of body weights of all mice in each treatment group.

Supplemental Figure 6

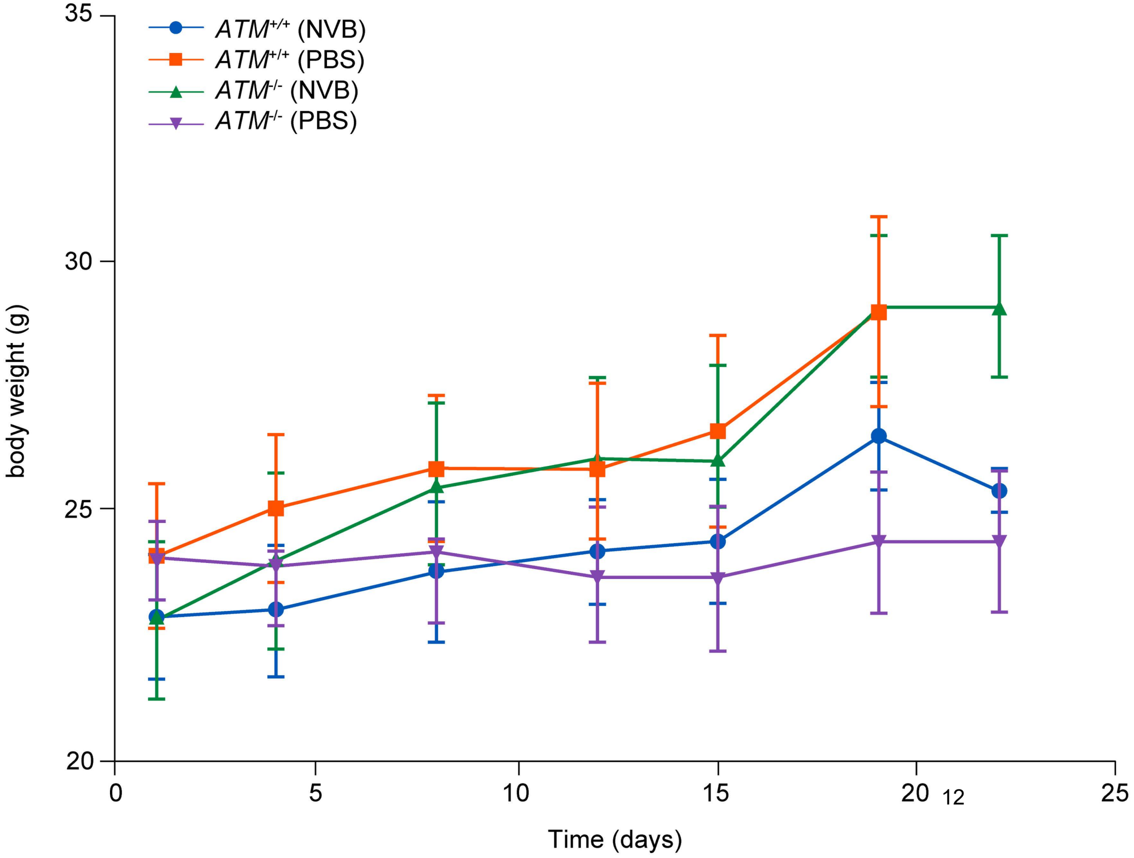

Supplemental Figure 7: (A) Assessment of cyclin D1 in non Hodgkin lymphoma (MZL n=1, FL n=1, CLL n=1 and MCL n=5). **(B)** Assessment of ATM and cyclin D1 through immunoblotting in primary MCL patients (n=28). Squires represent patients with ATM deficiency. * Represent patients who were not included in the POLQ mRNA expression assessment in Figure 7B or cell viability assessment with POLΘ inhibition in Figure 7C due to low number of cells in the primary sample. MZL: Marginal zone lymphoma, FL: Follicular lymphoma, CLL: Chronic lymphocytic leukemia, MCL: Mantle cell lymphoma.

Supplemental Figure 7

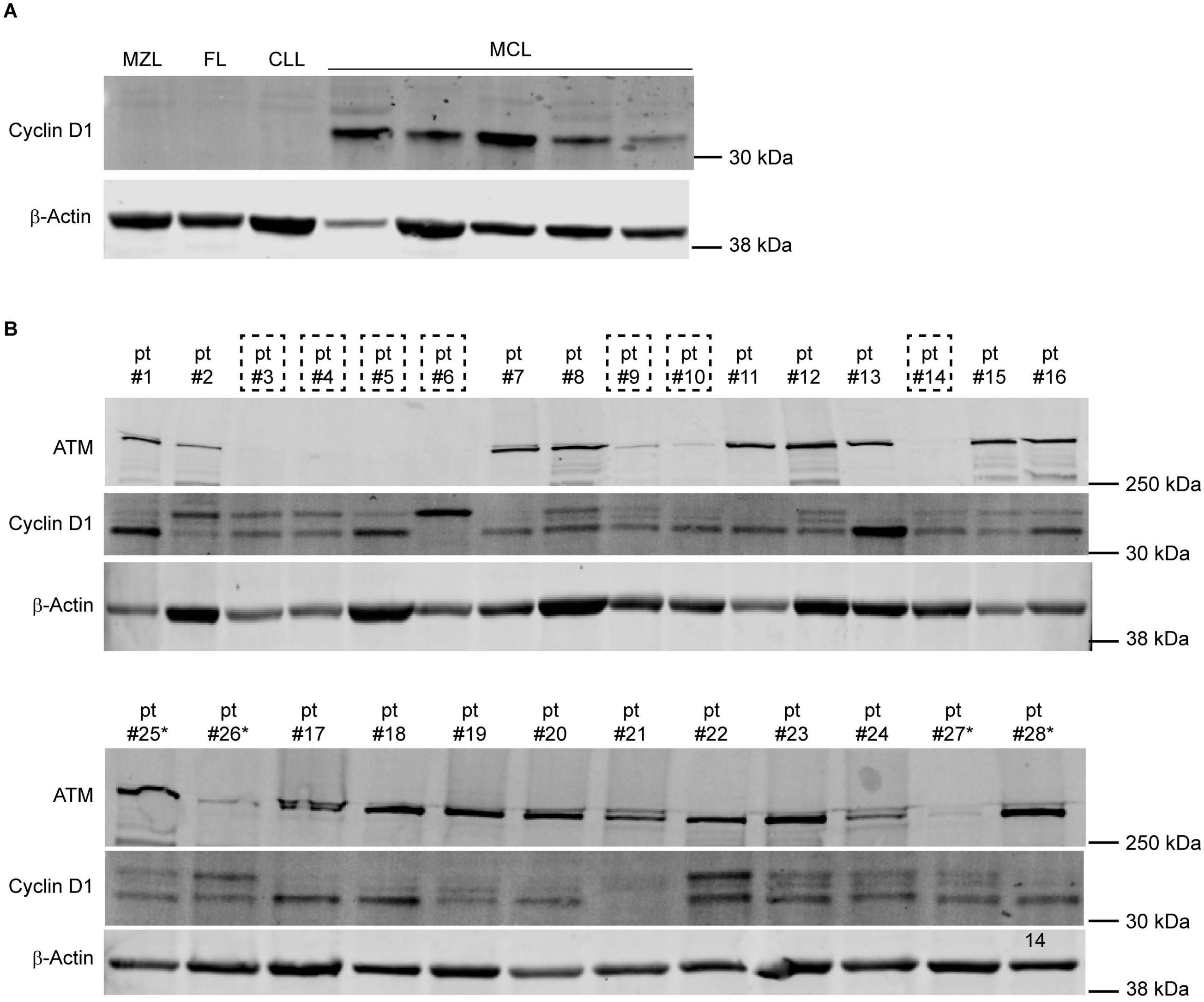

Supplemental Table 1: Guide RNA used for CRISPR knock-out, knock-in and interference experiments

**Gene Name**	**Guide RNA sequence**
CRISPR KO	
POLQ (sgRNA)	1. TCTGATCAATCGCCTCATAG<br>2. CTGACTCCAAAAGCGGTACA<br>3. GCATGTACTAGAATGTAACA<br>4. TGCCCGGAAGGCAGTGGATG
ATM (sgRNA)	1. TTTAAGCATATCATAGACCT<br>2. ATATGTGTTACGATGCCTTA<br>3. CTTCTACCCCAACAGCGACA<br>4. TTATTCCAGAAAGCCAAGGT
NT (sgRNA)	1. CGCUUCCGCGGCCCGUUCAA
CRISPR KI	
POLQ (sgRNA)	1. TTGCCATGAATCTTCTGCGT
**CRISPR interference**	
CCND1 (sgRNA)	1. CACCGTGCCAACCTCCTCAACGACC<br>2. CACCGCATTTGAAGTAGGACACCGA<br>3. CACCGGAGCTGGTGTTCCATGGCTG<br>4. CACCGGCAGAAGCGAGAGCCGAGCG
