# Supplementary material for: Cyclin D1 overexpression induces replication stress and microhomology-mediated end-joining dependence in mantle cell lymphoma
Source: J Clin Invest. 2025 Jul 3;135(17):e193006. doi: 10.1172/JCI193006 (PMC12404763; doi:10.1172/JCI193006)

Full unedited blot/gel for Figure 1C

Cyclin D1 U2OS

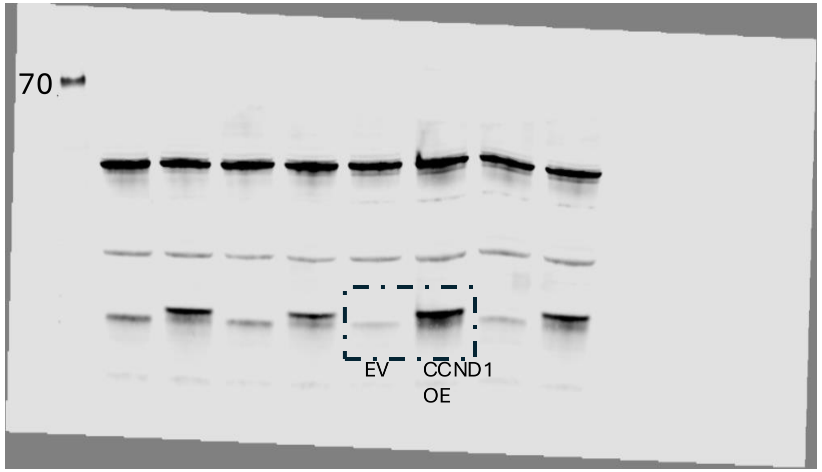

γ-H2AX

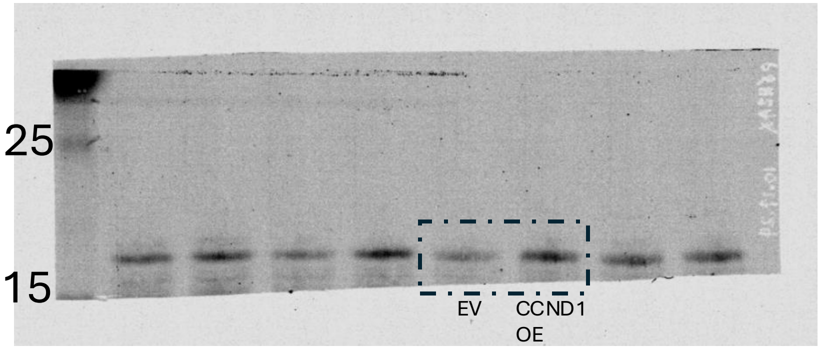

Actin U2OS

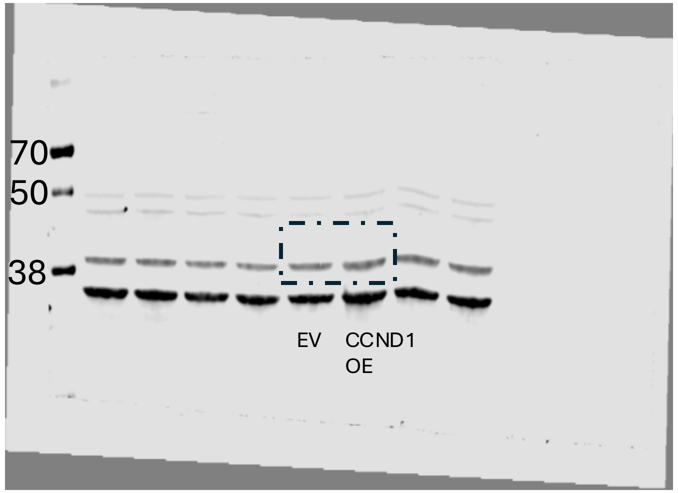

Full unedited blot/gel for Figure 2A

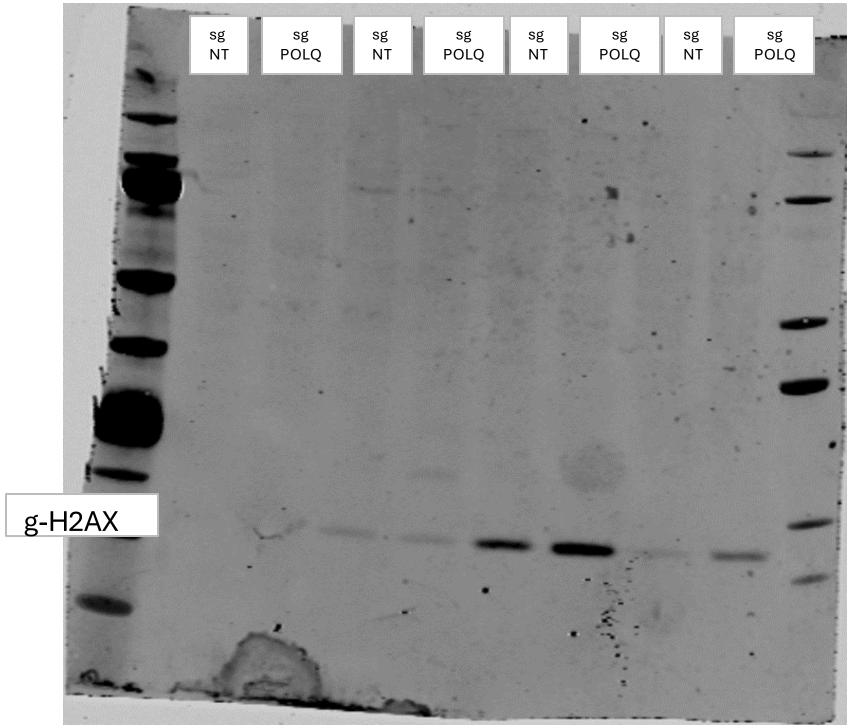

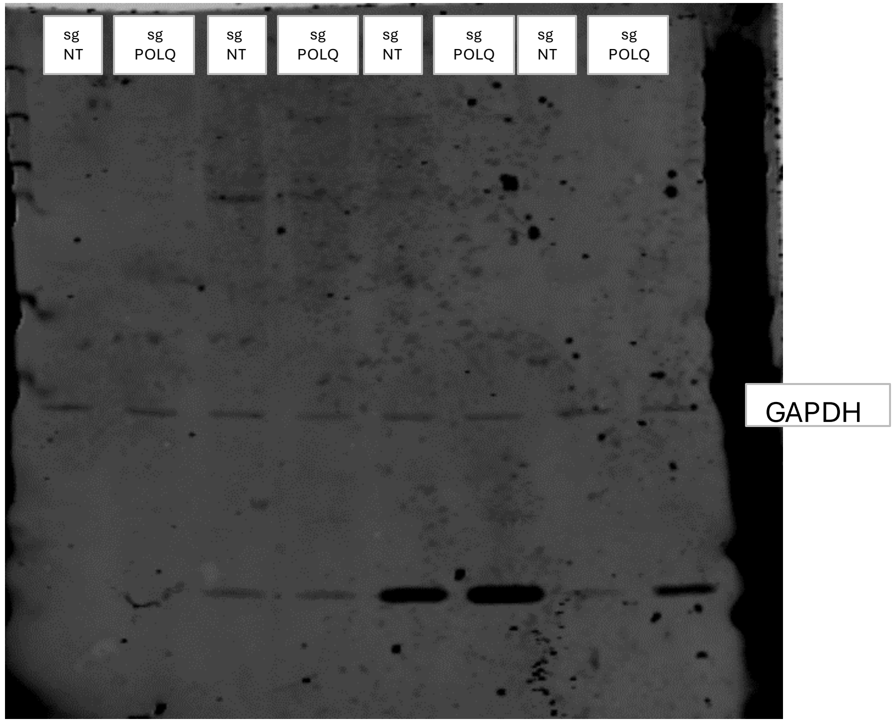

Full unedited blot/gel for Figure 2E

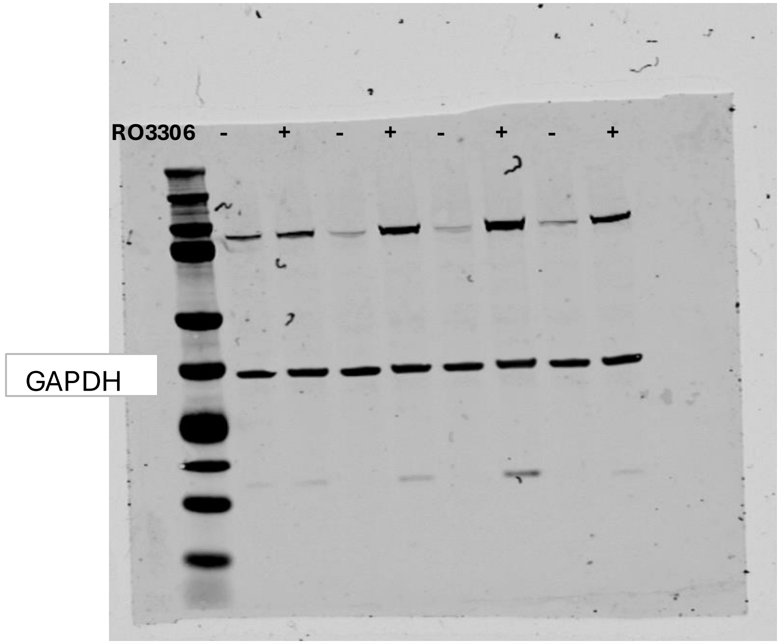

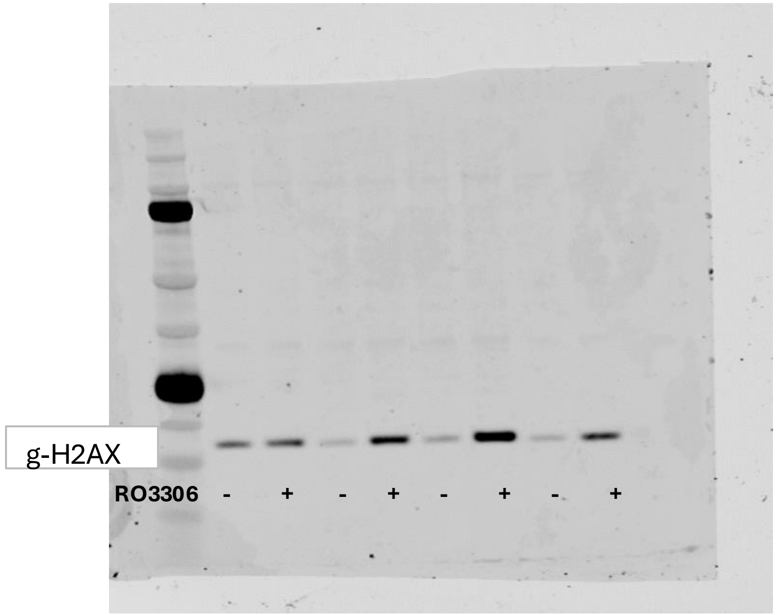

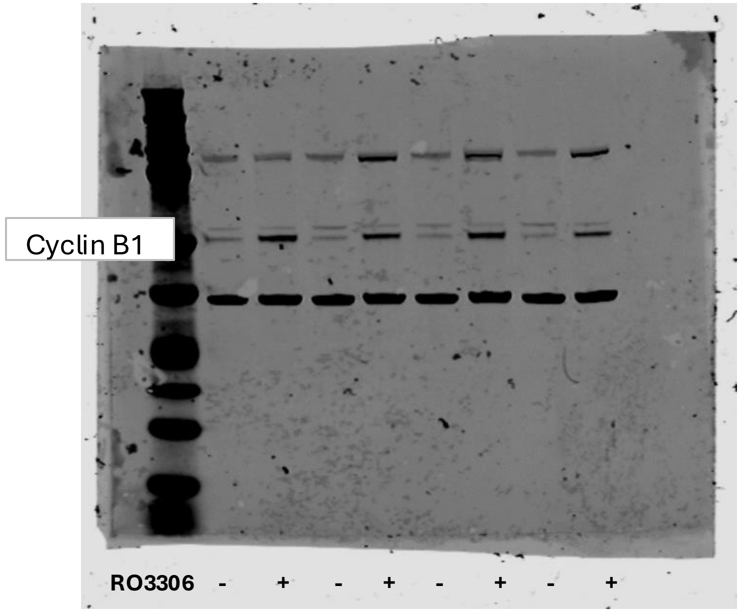

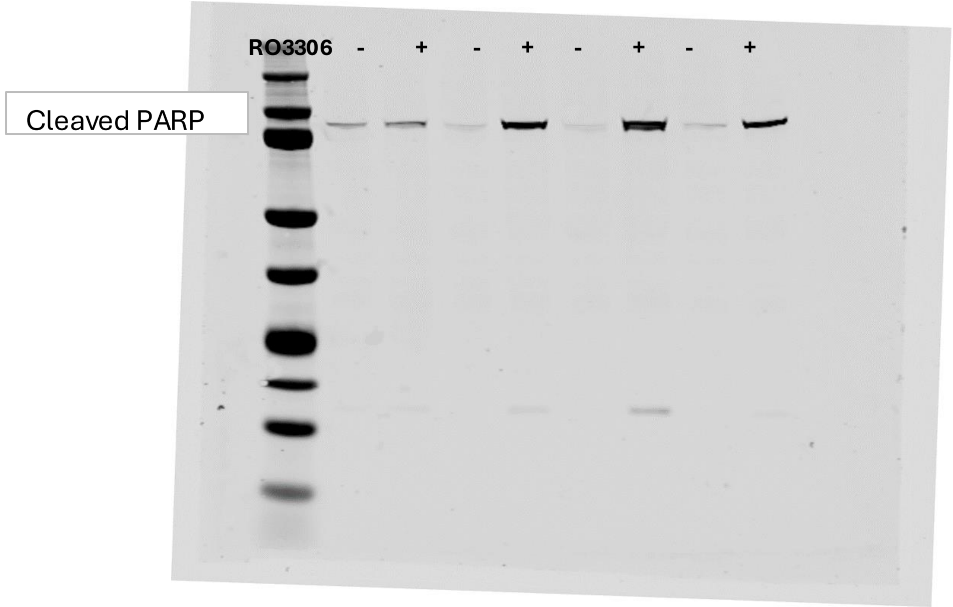

Full unedited blot/gel for Figure 3A

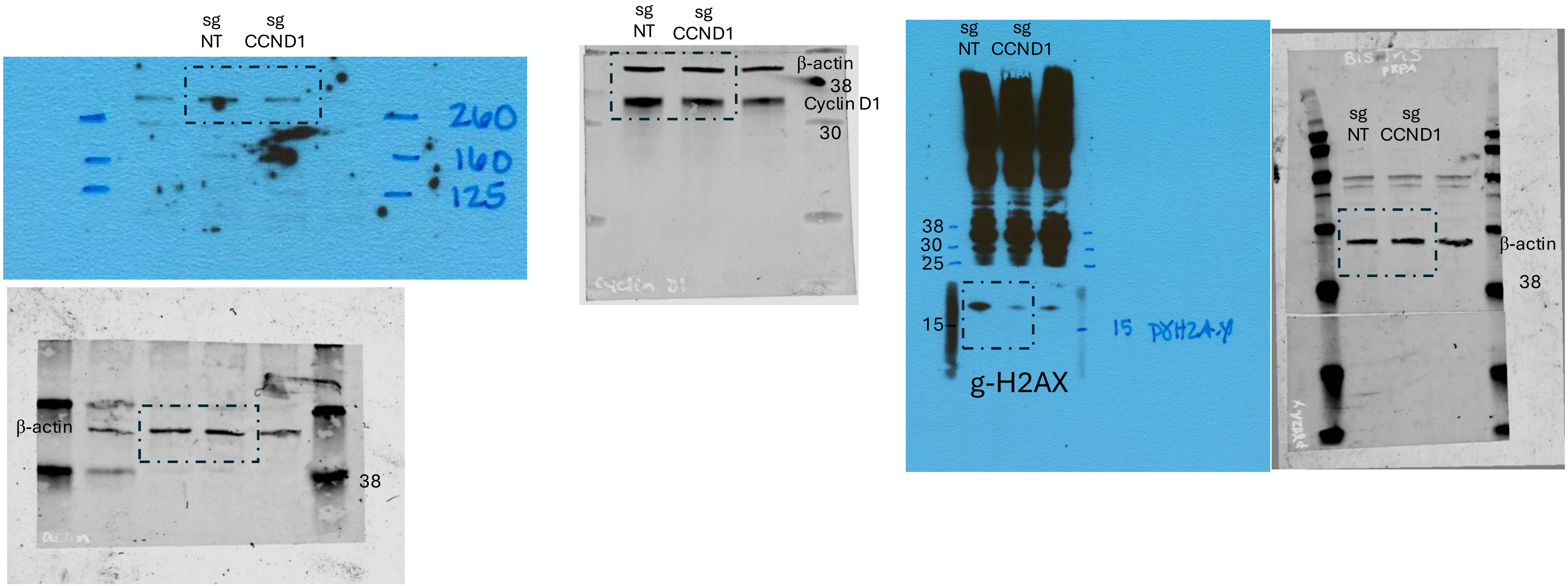

Full unedited blot/gel for Figure 4D

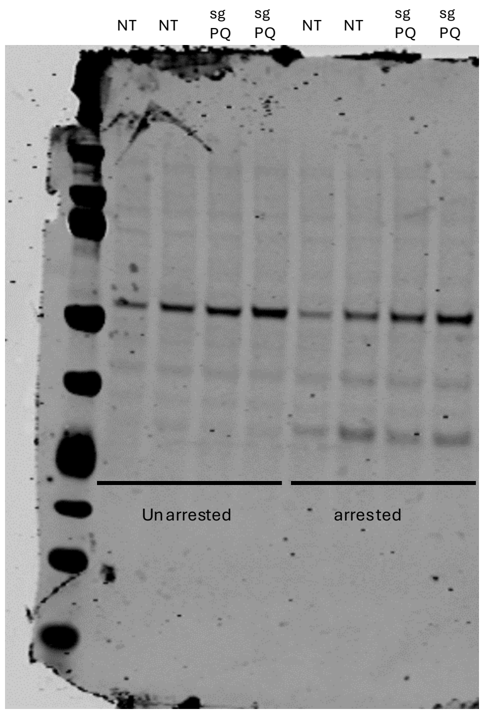

p-CHK1

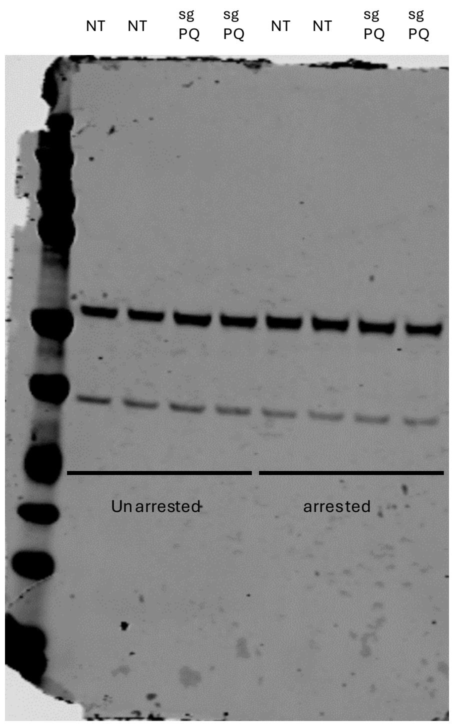

CHK1

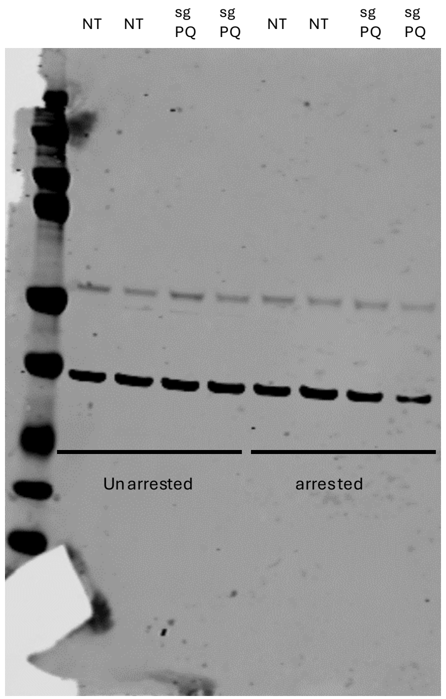

GAPDH

Full unedited blot/gel for Figure 5A

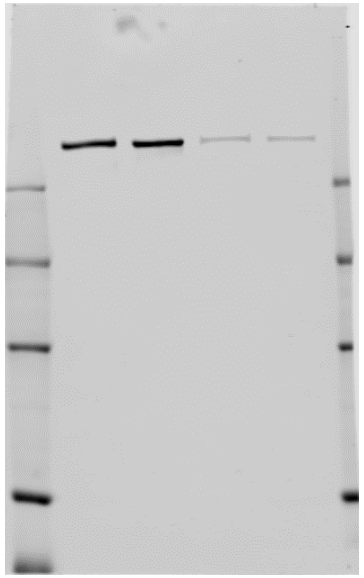

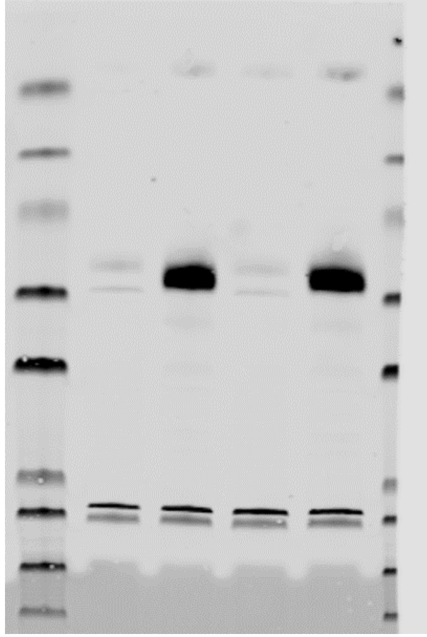

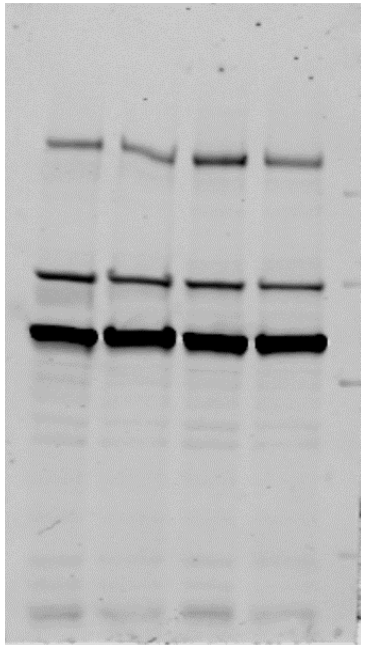

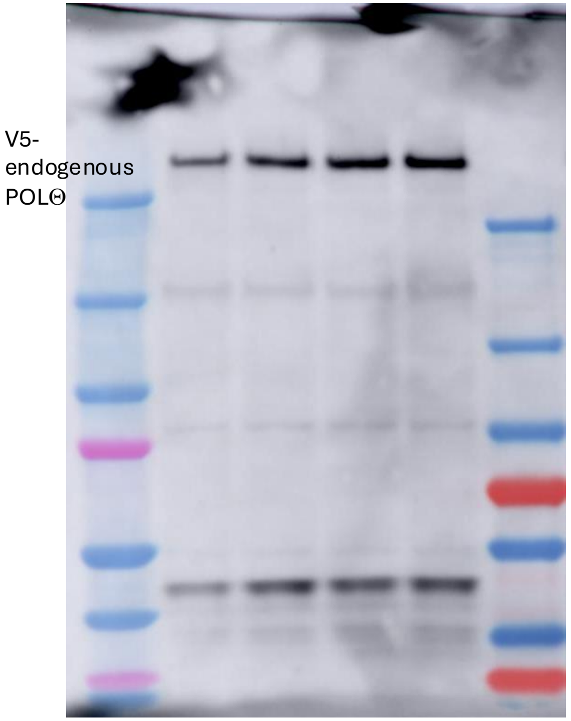

Full unedited blot/gel for Supplemental Figure 1B

Cyclin D1

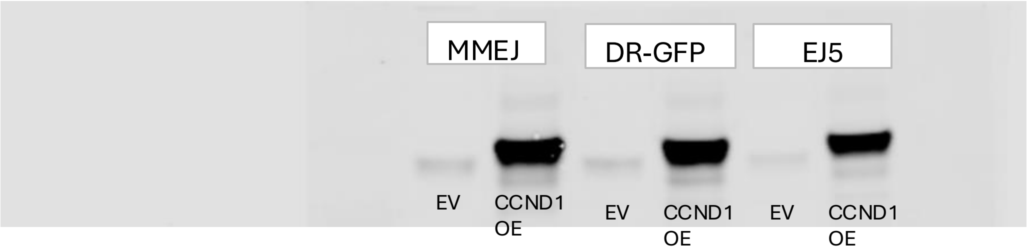

GAPDH

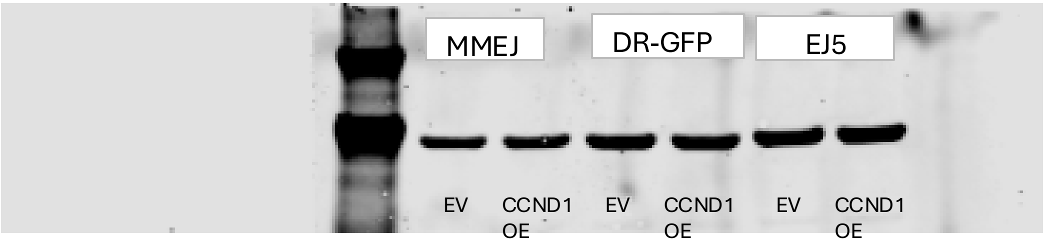

Full unedited blot/gel for Supplemental Figure 1E

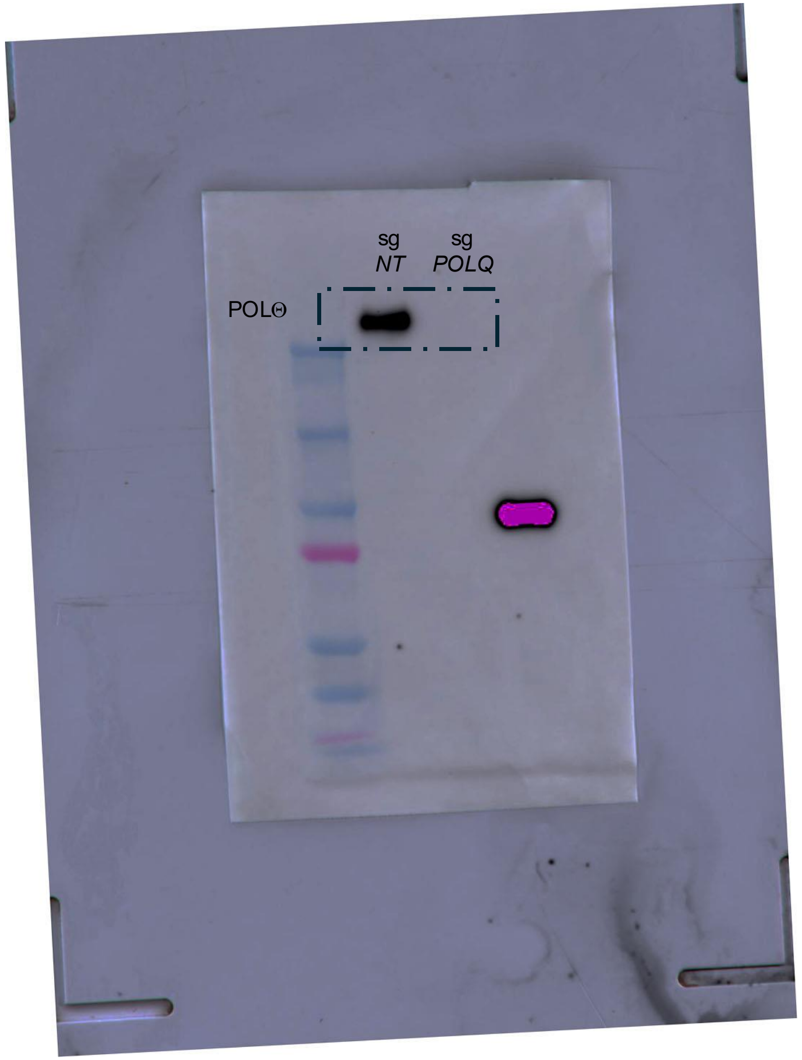

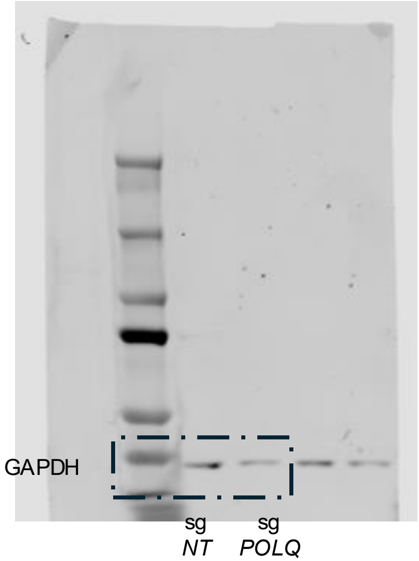

Full unedited blot/gel for Supplemental Figure 2A

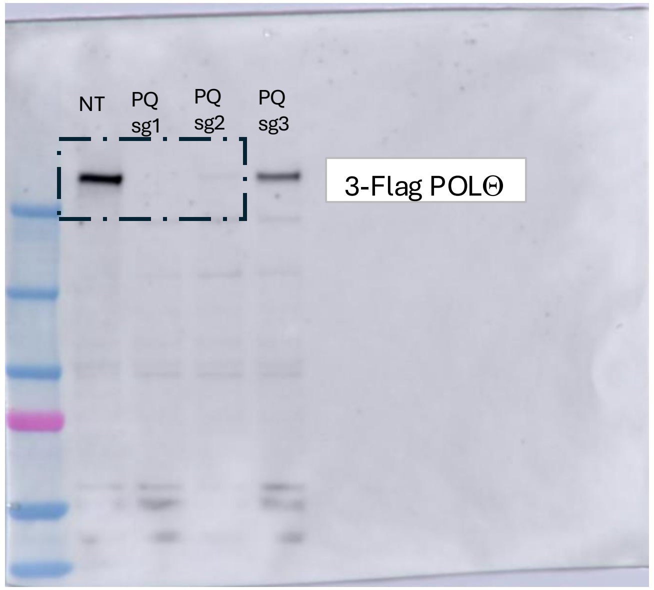

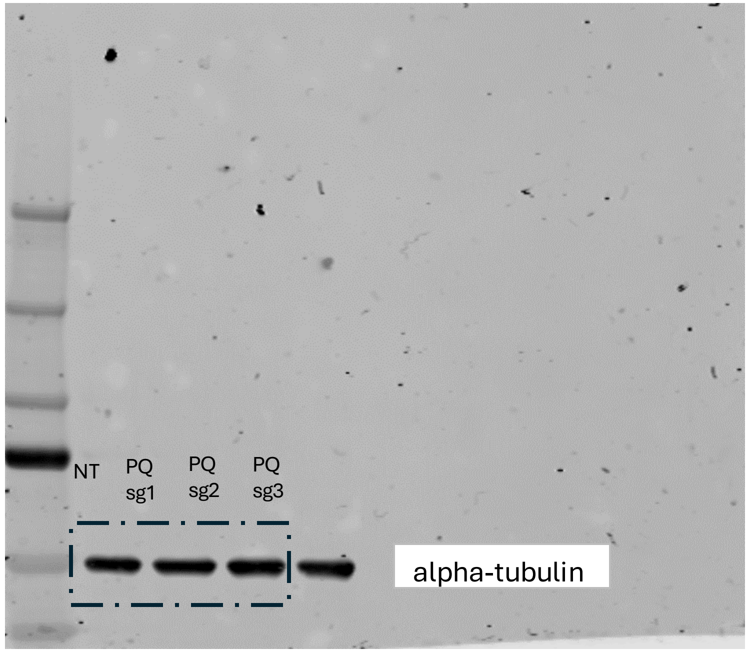

Full unedited blot/gel for Supplemental Figure 3B

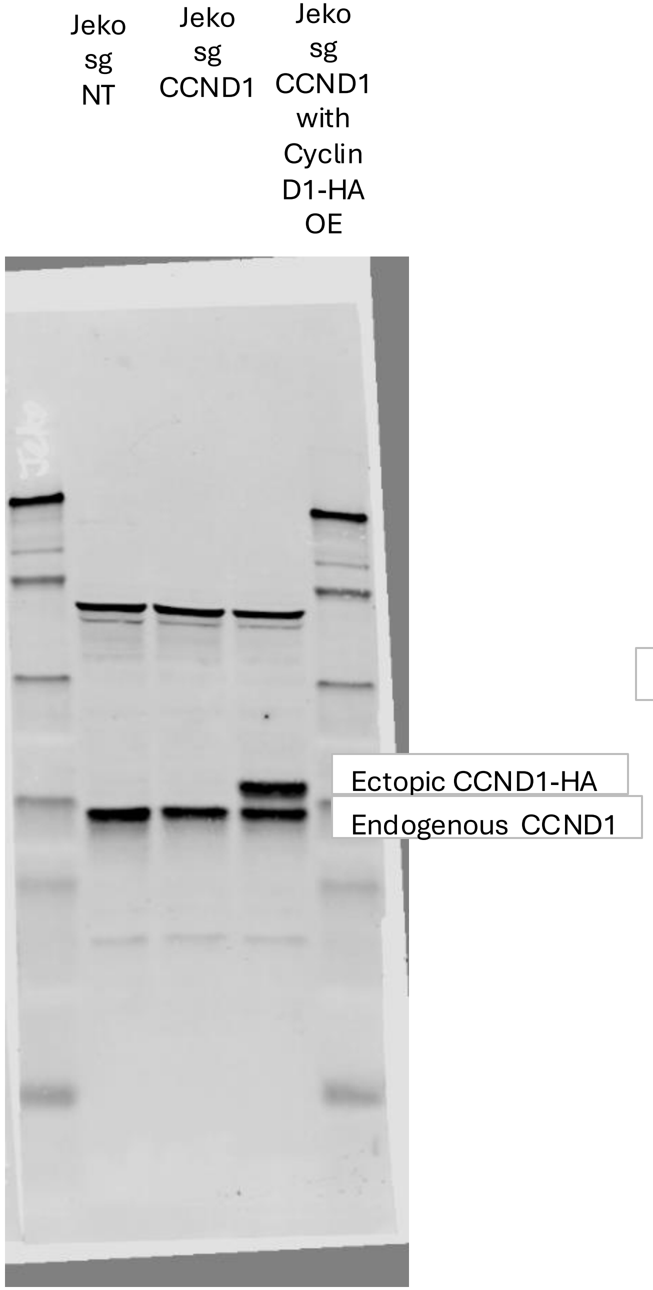

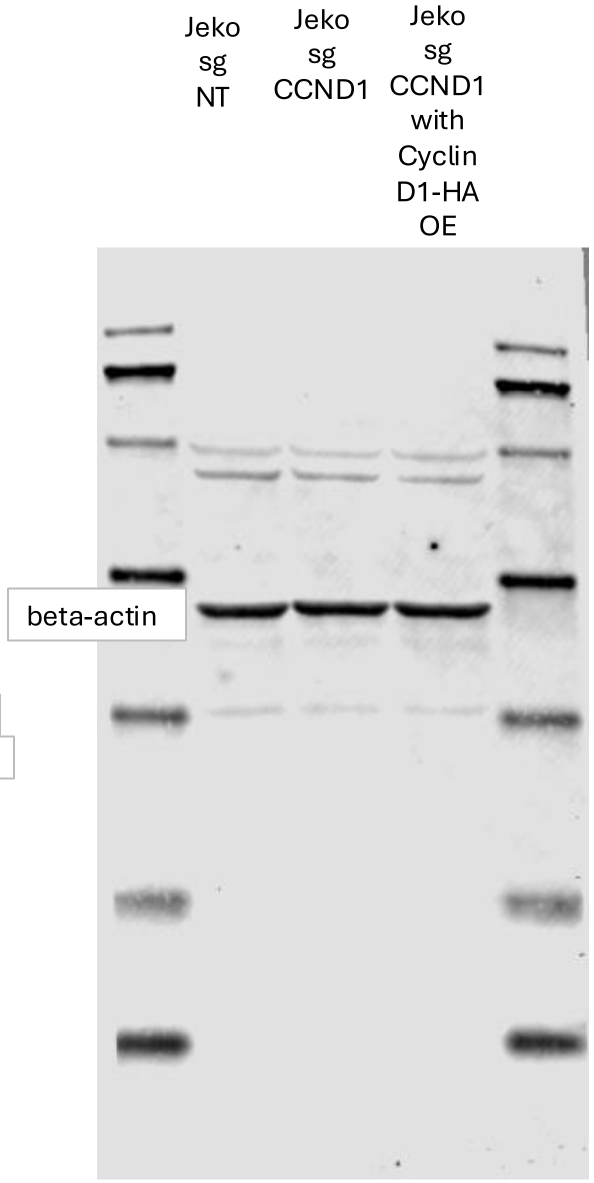

Full unedited blot/gel for Supplemental Figure 3C

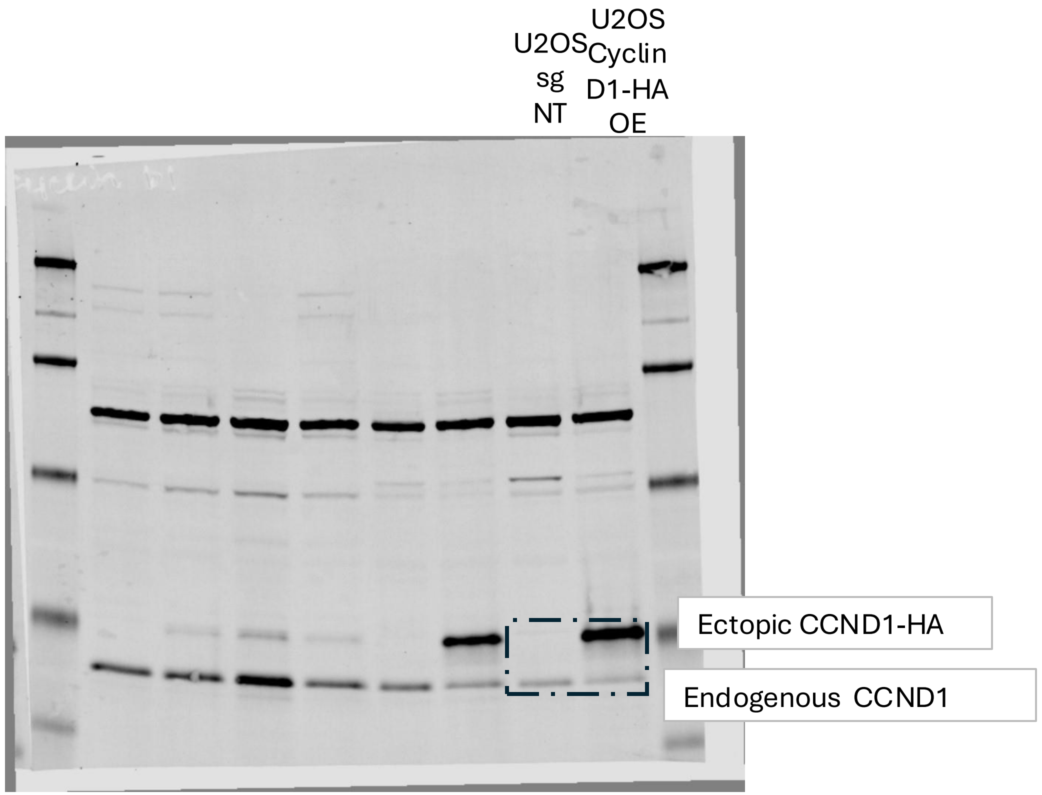

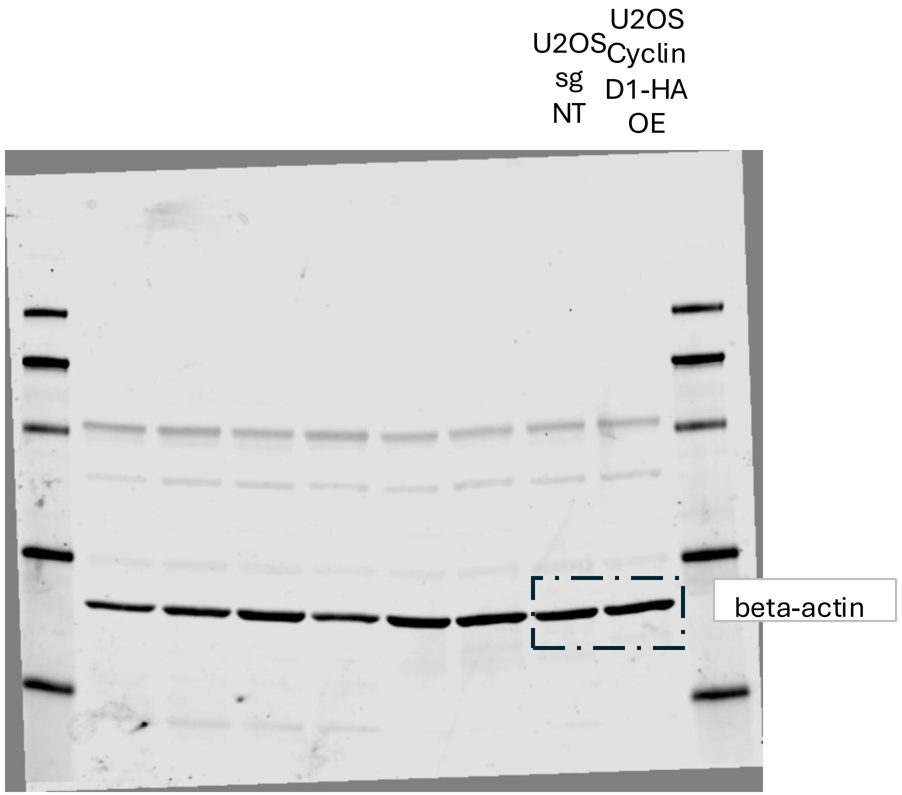

Full unedited blot/gel for Supplemental Figure 4 A-C

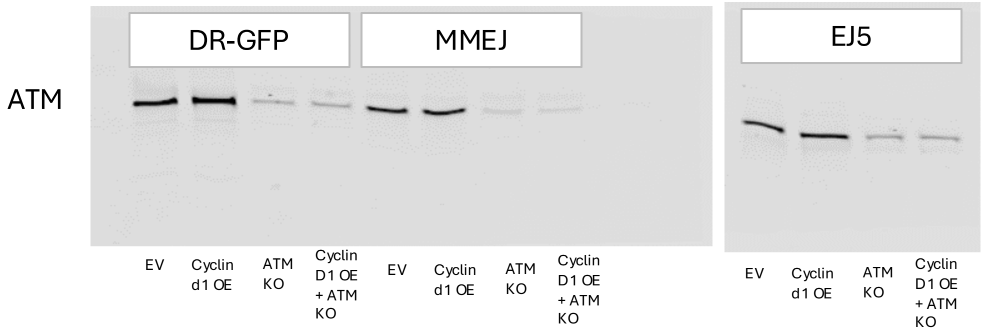

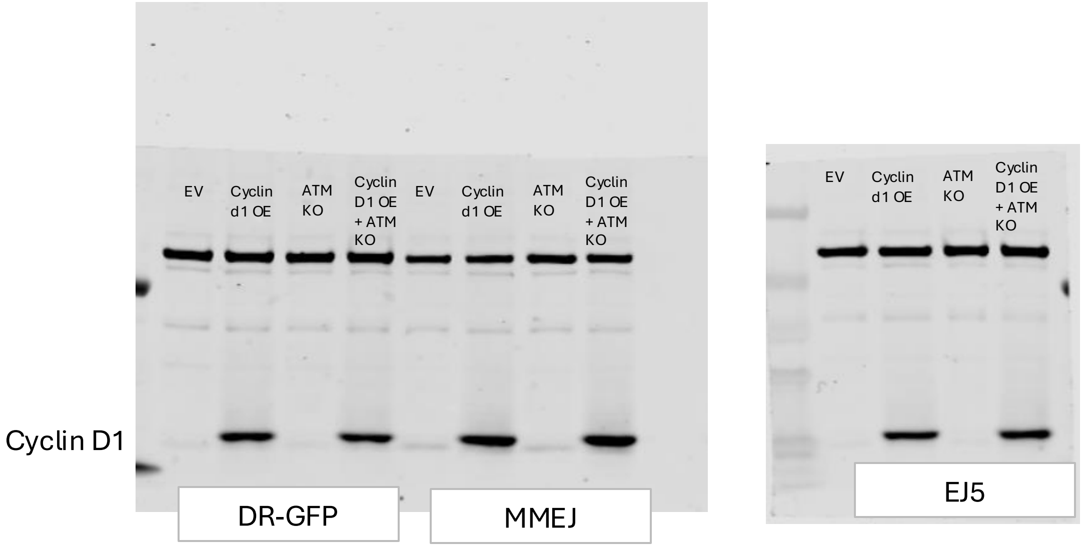

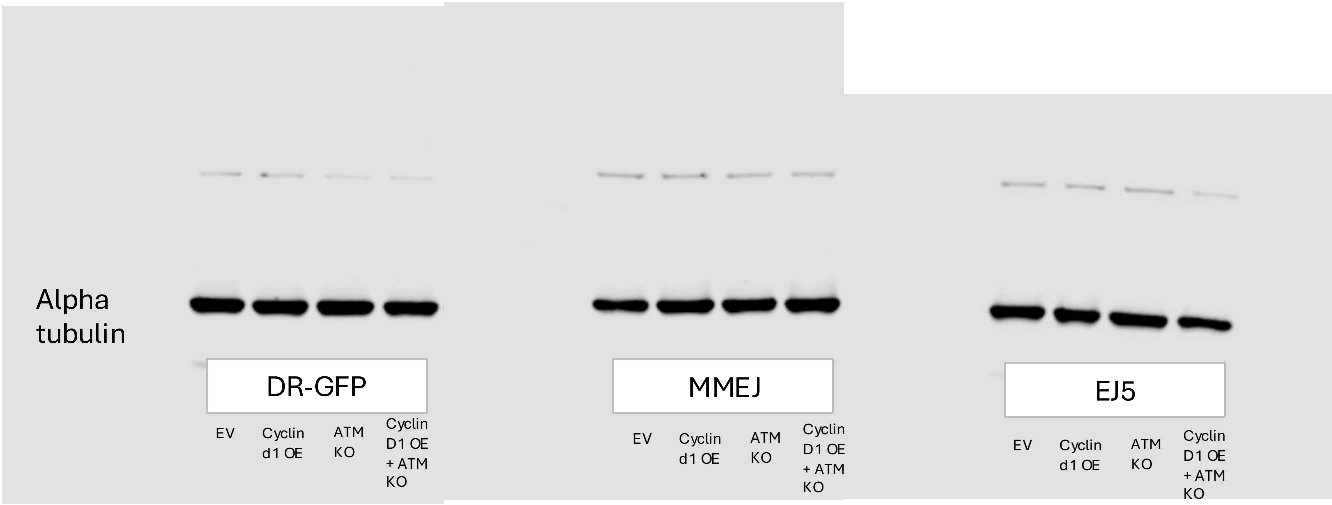

Full unedited blot/gel for Supplemental Figure 4D

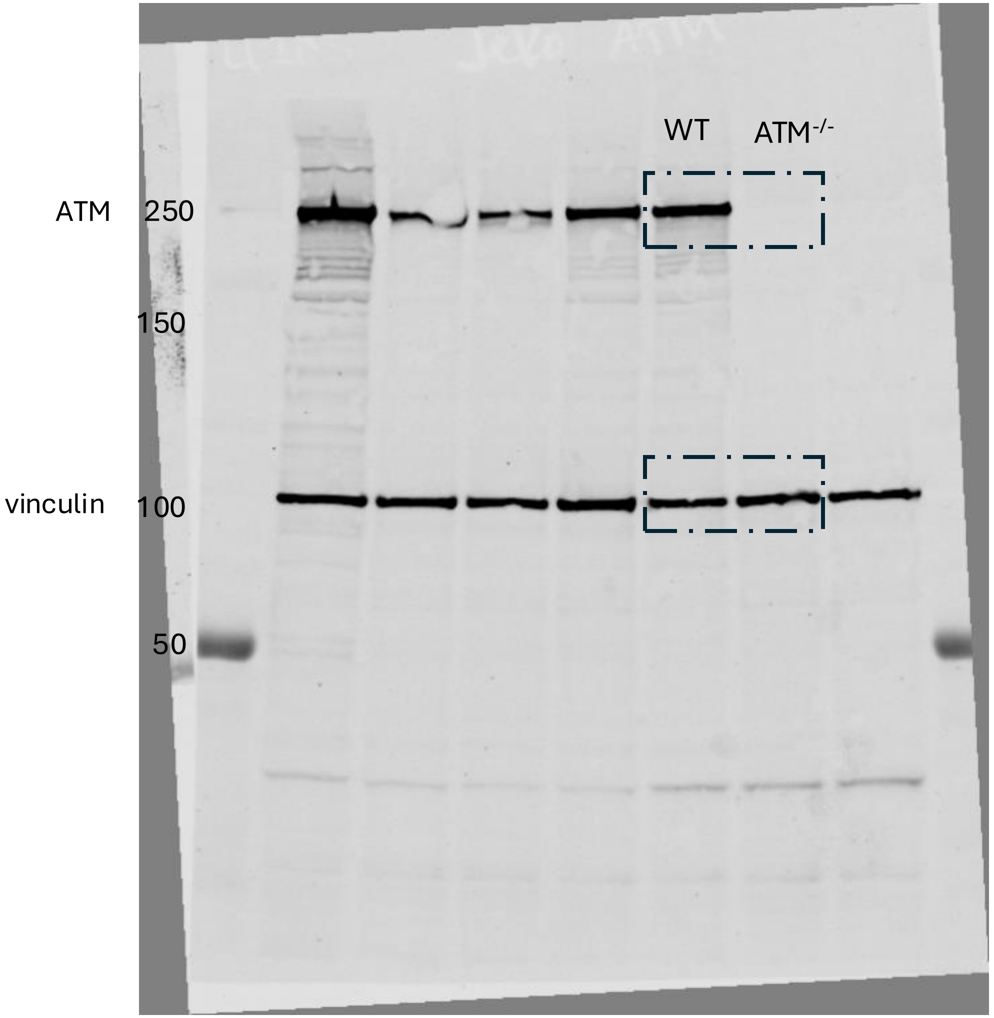

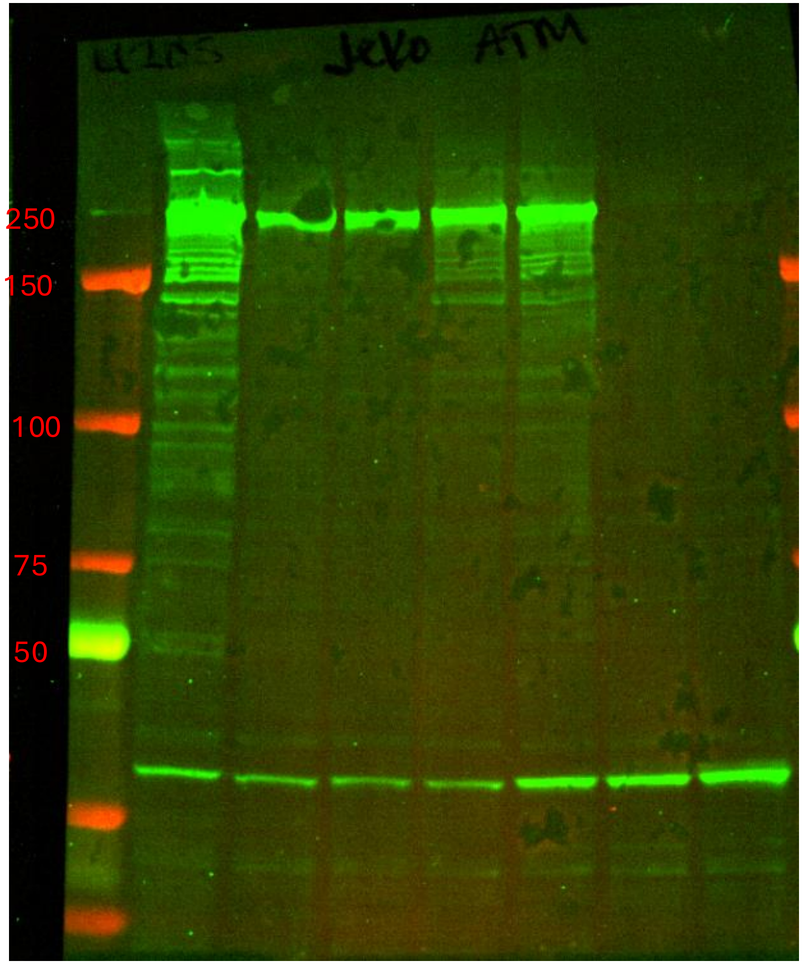

Full unedited blot/gel for Supplemental Figure 5B

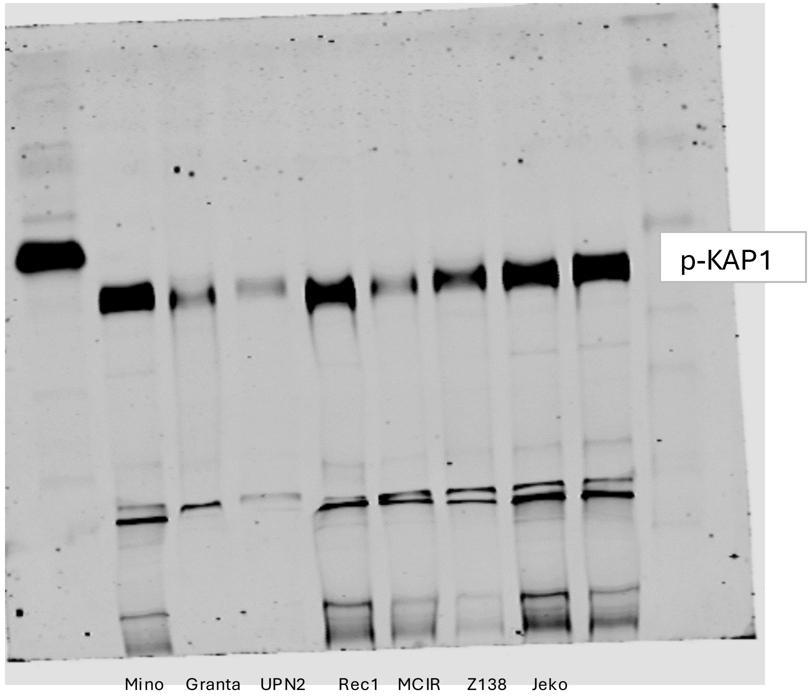

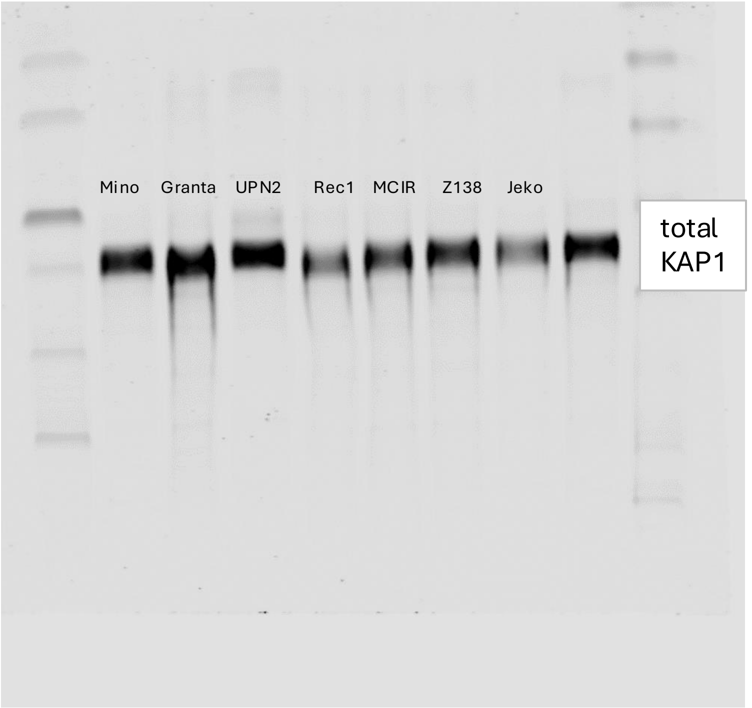

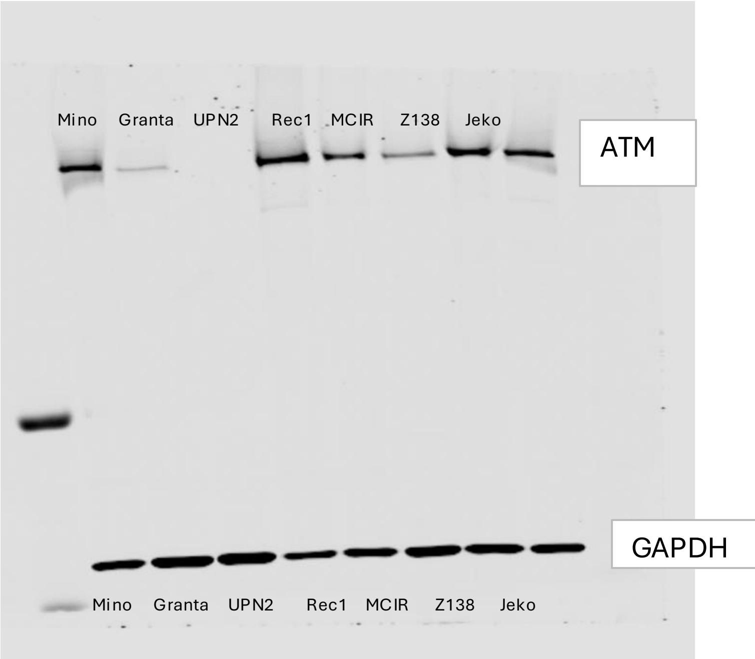

Full unedited blot/gel for Supplemental Figure 7A

Cyclin D1

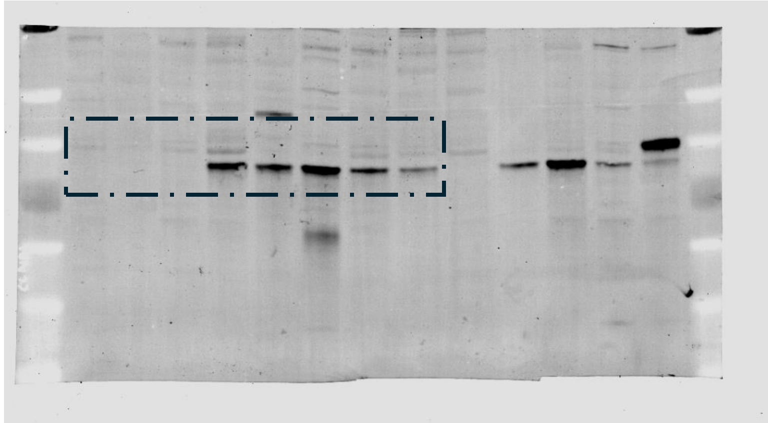

β-actin

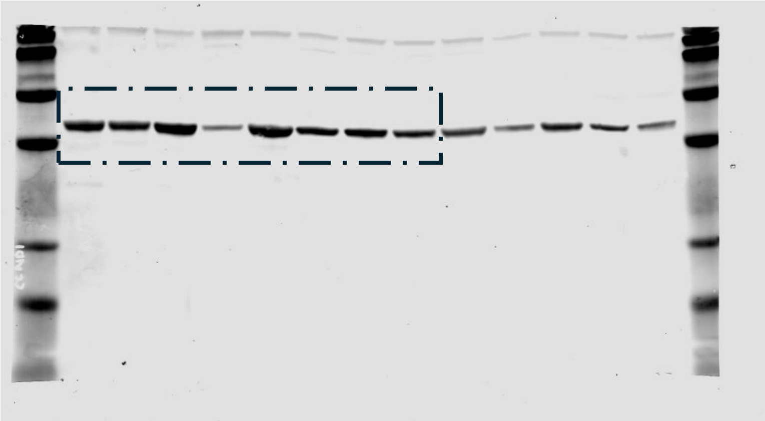

Full unedited blot/gel for Supplemental Figure 7B

ATM

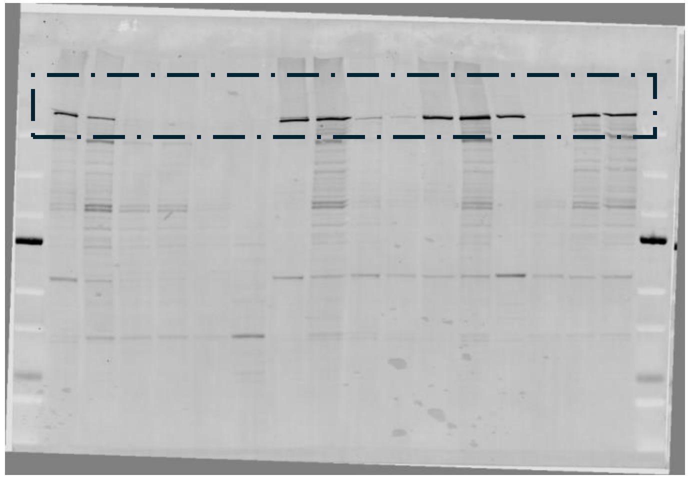

ATM

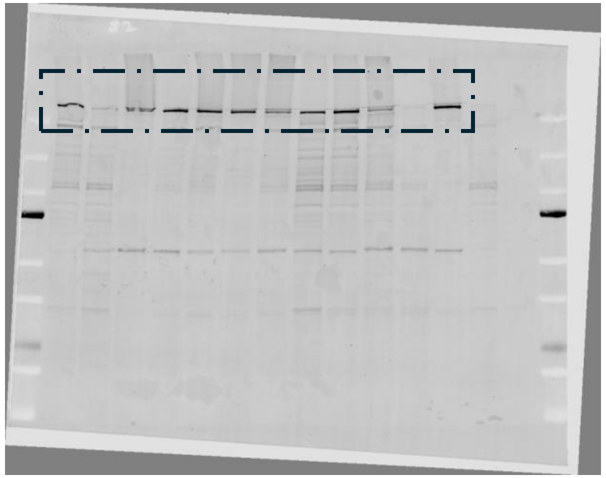

Cyclin D1

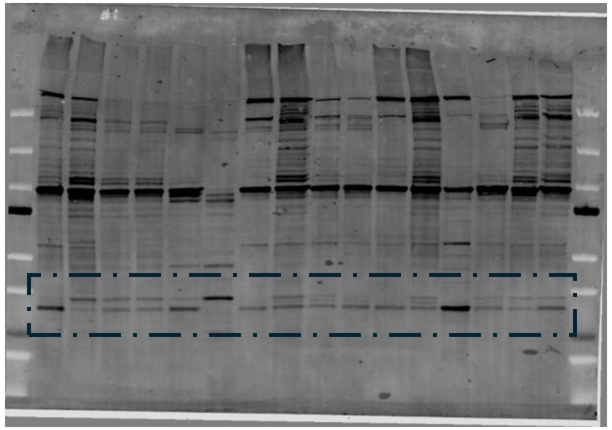

Cyclin D1

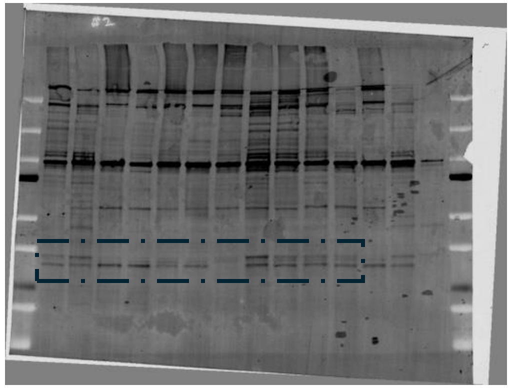

Actin

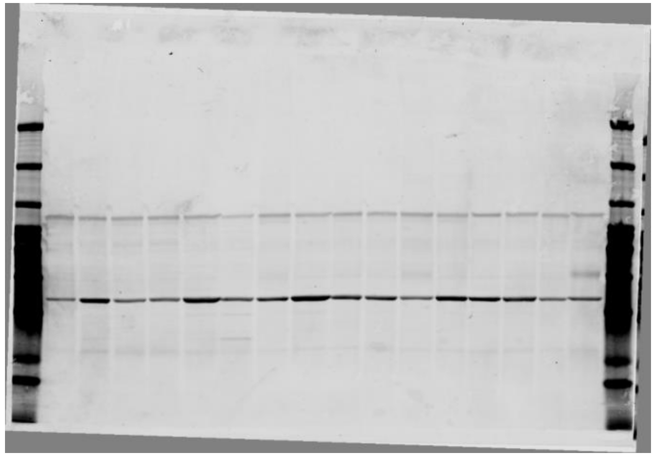

Actin

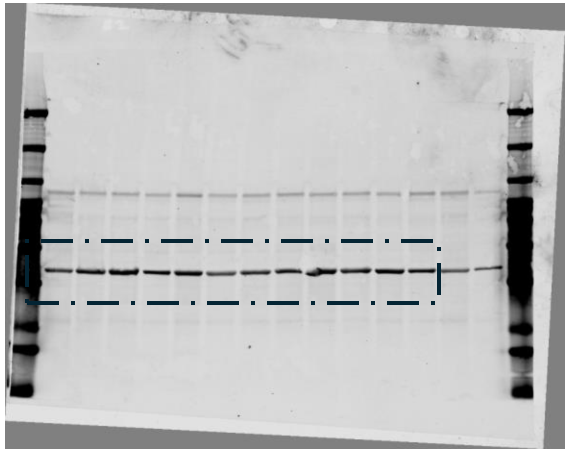

Supplement: Unedited blot and gel images [file jci-135-193006-s207.pdf]
